# Cross‐Regulation Between DNA Methylation and Circadian Clock Reprogramming in Ovarian Cancer: Mechanistic Insights, Biomarker Potential, and Therapeutic Implications

**DOI:** 10.1002/ijc.70704

**Published:** 2026-08-19

**Authors:** Min Xing, Chang Zhou, Yi Tang, Shuhui Xu, Shasha Zhou, Weiai Liu, Huiping Liu

**Affiliations:** ^1^ Hunan University of Chinese Medicine Changsha Hunan China; ^2^ Hinye Pharmaceutical Co. Ltd. Changsha Hunan China; ^3^ The Second Affiliated Hospital of Hunan University of Chinese Medicine Changsha Hunan China

**Keywords:** circadian rhythm, DNA methylation, epigenetic reprogramming, ovarian cancer

## Abstract

Early diagnosis remains challenging, recurrence rates remain high, and platinum resistance frequently develops in ovarian cancer (OC), collectively representing major barriers to long‐term patient survival. DNA methylation (DNAm), as a relatively stable yet dynamically modifiable epigenetic signature, can capture alterations in tumor states and evolutionary trajectories associated with therapeutic pressure. Meanwhile, the circadian rhythm system regulates multiple cancer‐related processes, including DNA damage repair, cell‐cycle progression, metabolic adaptation, and immune microenvironment remodeling, thereby influencing OC development and treatment responses. Accumulating evidence suggests the existence of a bidirectional interplay between DNAm landscapes and the circadian clock network in OC. This review summarizes the emerging mechanisms underlying the crosstalk between DNAm and circadian reprogramming in OC, and further discusses their potential applications in early detection, prognostic stratification, dynamic monitoring of platinum resistance, and chronotherapy‐based therapeutic strategies. Although current findings highlight the promising translational potential of the DNAm–circadian axis, its clinical implementation remains constrained by tumor heterogeneity, variations in sampling time points, cellular composition complexity, and insufficient validation across experimental models and clinical cohorts. Future integration of time‐resolved sampling approaches, liquid biopsy platforms, single‐cell and spatial epigenomic technologies, as well as prospective clinical trials evaluating circadian‐guided interventions, may further define the translational boundaries and clinical utility of DNAm–circadian coupling in precision diagnosis and treatment of OC.

## Graphical abstract

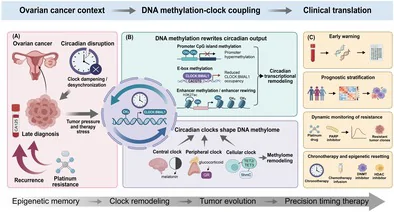

**Abbreviations**
AKT: protein kinase B
ARNTL: aryl hydrocarbon receptor nuclear translocator‐like protein
BMAL1: brain and muscle ARNT‐like 1
BRCA1: breast cancer 1
BRCA2: breast cancer 2
CA125: cancer antigen 125
CCGs: clock‐controlled genes
cfDNA: cell‐free DNA
CLOCK: circadian locomotor output cycles kaput
CpG: cytosine–phosphate–guanine
CRY: cryptochrome circadian regulator
CRY1: cryptochrome circadian regulator 1
ctDNA: circulating tumor DNA
DNAm: DNA methylation
DNMT: DNA methyltransferase
E‐box: enhancer‐box
EMT: epithelial–mesenchymal transition
FIGO: International Federation of Gynecology and Obstetrics
GEO: gene expression omnibus
GnRH: gonadotropin‐releasing hormone
GR: glucocorticoid receptor
HGSOC: high‐grade serous ovarian cancer
HIF‐1α: hypoxia‐inducible factor‐1 alpha
HPO: hypothalamic–pituitary–ovarian axis
HR: homologous recombination
HRD: homologous recombination deficiency
LH: luteinizing hormone
MBDs: methyl‐CpG‐binding domain proteins
MeCP2: methyl‐CpG‐binding protein 2
MYC: MYC proto‐oncogene
NR3C1: nuclear receptor subfamily 3 group C member 1
OC: ovarian cancer
P4: progesterone
PARP: poly(ADP‐ribose) polymerase
PARPi: PARP inhibitor
PD: pharmacodynamic
PER: period circadian protein
PER2: period circadian regulator 2
PI3K: phosphoinositide 3‐kinase
PK: pharmacokinetic
RAD51C: RAD51 paralog C
SCN: suprachiasmatic nucleus
SIRT1: sirtuin 1
STAR: steroidogenic acute regulatory protein
TCGA: The Cancer Genome Atlas
TET: ten‐eleven translocation dioxygenase
TF: transcription factor
TIMELESS: timeless circadian regulator
TTFL: transcription–translation feedback loop
5hmC: 5‐Hydroxymethylcytosine
5mC: 5‐Methylcytosine

## 1 Introduction

Ovarian cancer (OC) is one of the most lethal malignancies of the female reproductive system, with poor outcomes largely driven by delayed diagnosis, recurrence, and drug resistance [1]. Current surveillance strategies based on imaging and CA125 remain inadequate for detecting occult disease, minimal residual disease, and emerging resistance [2, 3]. Although cytoreductive surgery followed by platinum‐based chemotherapy provides initial responses, most patients eventually develop recurrence through complex evolutionary processes involving DNA damage repair, metabolic adaptation, microenvironment remodeling, immune evasion, and epigenetic reprogramming rather than isolated genetic events [4, 5, 6]. Therefore, multidimensional molecular approaches are required to capture tumor‐state transitions and treatment adaptation.

DNA methylation (DNAm) represents a clinically relevant epigenetic layer in OC [7, 8]. Aberrant DNAm alterations occur early during tumorigenesis and regulate promoters, enhancers, DNA repair pathways, drug sensitivity, and cellular plasticity [9, 10, 11, 12]. In HGSOC, extensive methylation remodeling is associated with therapeutic response, resistance, and prognosis [11, 13, 14]. Due to its stability, detectability, and reversibility, DNAm profiling in tumor tissues, cfDNA, and ctDNA holds potential for early detection, monitoring, and molecular stratification [15, 16, 17].

The circadian clock provides an additional temporal regulatory framework for OC biology by controlling cell cycle, DNA repair, metabolism, apoptosis, and immunity [18, 19, 20, 21]. Dysregulated clock components are associated with proliferation, cisplatin sensitivity, prognosis, and immune characteristics [20, 21, 22, 23].

Importantly, DNAm and circadian networks are interconnected through bidirectional regulation [24]. Epigenetic silencing of circadian genes, such as ARNTL (BMAL1) promoter methylation, impairs tumor suppression, cisplatin sensitivity, and circadian transcriptional activity [25, 26, 27], whereas circadian mechanisms can regulate dynamic DNAm patterns [28]. This review discusses the emerging interplay between DNAm and circadian regulation in OC and their potential implications for diagnosis, prognosis, resistance monitoring, and therapeutic optimization.

## 2 Circadian Clock Networks in Normal Ovarian Function and OC

### 2.1 Transcription–Translation Feedback Loops (TTFL) and Epigenetic Interfaces of Core Clocks

The circadian clock is an intrinsic timing system that coordinates mammalian physiological and behavioral processes through an approximately 24‐h cycle and is synchronized by external zeitgebers such as light and feeding patterns [29]. Circadian rhythms are generated by the transcription–TTFL, which is closely integrated with chromatin accessibility, histone modifications, and rhythmic transcription factor binding [30, 31].

The CLOCK–BMAL1 (ARNTL) heterodimer activates PER, CRY, and clock‐controlled genes (CCGs) through E‐box binding, while PER/CRY‐mediated negative feedback regulates circadian period, amplitude, and stability [32, 33, 34, 35, 36, 37, 38, 39, 40]. REV‐ERB and ROR nuclear receptors further stabilize BMAL1 expression and connect circadian regulation with metabolism [41, 42].

Circadian transcription is highly dependent on epigenetic regulation. CLOCK‐mediated histone acetylation, p300/CBP recruitment, and SIRT1‐dependent deacetylation collectively regulate rhythmic chromatin states and transcriptional activity [43, 44, 45]. DNAm provides an additional stable regulatory layer that can maintain persistent circadian alterations [46, 47]. In cancer, BMAL1/ARNTL promoter CpG hypermethylation induces transcriptional repression, whereas BMAL1 restoration exerts tumor‐suppressive effects [48, 49]. DNAm can impair transcription factor binding, recruit methyl‐CpG‐binding proteins, and promote repressive chromatin formation [50, 51]. Moreover, methylation of CpG‐containing E‐box motifs directly reduces CLOCK binding and alters TTFL outputs [52, 53]. Thus, aberrant DNAm may stabilize circadian disruption and contribute to circadian reprogramming in malignancies such as OC.

### 2.2 Ovarian Temporal Homeostasis: From Steroidogenesis to the Ovulatory Window

The ovary is not merely an endocrine target organ; steroidogenesis, follicular maturation, and ovulation require precise temporal coordination between circadian rhythms and estrous‐cycle regulation. This temporal homeostasis depends on integration of central circadian signals and ovarian peripheral clocks, which regulate steroidogenic activity and gonadotropin‐driven events [54].

Circadian control of ovarian steroidogenesis involves rhythmic regulation of key steroidogenic genes. STAR, a clock‐controlled gene containing E‐box elements, interacts with CLOCK to establish circadian expression patterns during follicular development [55, 56]. In human luteinized granulosa cells, BMAL1 expression correlates with STAR, CYP11A1, and CYP19A1, and BMAL1 manipulation alters steroidogenic networks, indicating a role for local ovarian clocks in endocrine regulation [57]. Thus, circadian mechanisms establish temporal patterns of ovarian activity, while estrous‐cycle signals further shape hormonal fluctuations [1, 58].

Reproductive function also depends on circadian gating within the hypothalamic–pituitary–ovarian (HPO) axis [59, 60]. Ovulation occurs within a restricted temporal window largely determined by circadian regulation of the preovulatory LH surge [54]. The SCN integrates circadian signals with estrogen feedback to regulate GnRH/LH secretion through mediators such as kisspeptin, while ovarian clocks modulate gonadotropin responsiveness and follicular–luteal transition [54, 61, 62, 63, 64, 65, 66, 67]. Conditional Bmal1 deletion in theca cells disrupts LH responsiveness, ovulation, and fertility, demonstrating the importance of ovarian clocks in reproductive timing [66]. Because ovulation involves inflammatory remodeling, oxidative stress, and DNA damage responses [68, 69], circadian disruption or epigenetic alterations may impair ovarian homeostasis and potentially contribute to epithelial injury and malignant transformation [70].

Collectively, ovarian temporal organization maintains reproductive homeostasis, and its disruption may link circadian dysregulation, epigenetic alterations, and disease progression (Figure 1).

**FIGURE 1:**
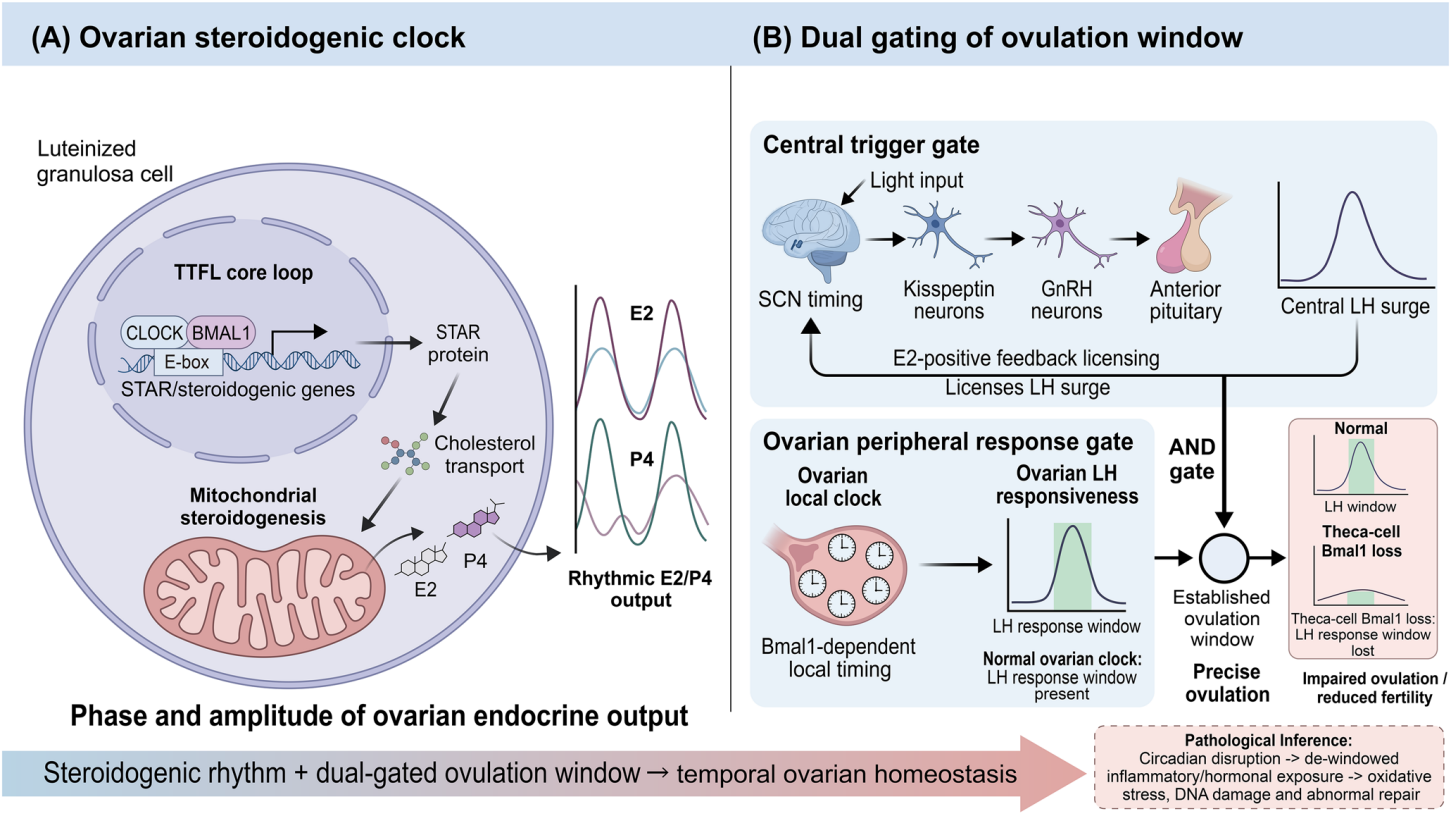
Circadian regulation of ovarian steroidogenesis and ovulation timing. (A) Ovarian steroidogenic clocks regulate rhythmic hormone production. CLOCK:BMAL1‐driven TTFL networks control steroidogenic gene expression, including STAR, promoting cholesterol transport, mitochondrial steroidogenesis, and rhythmic E2/P4 secretion in granulosa cells. (B) Dual circadian gating ensures precise ovulation timing. SCN‐driven central signals regulate LH surge initiation, while ovarian Bmal1‐dependent peripheral clocks establish LH responsiveness windows. Disruption of the ovarian clock impairs temporal coordination, leading to abnormal hormonal exposure, oxidative stress, and impaired ovarian homeostasis. BMAL1, brain and muscle ARNT‐like 1; Bmal1, brain and muscle ARNT‐like 1; CLOCK, circadian locomotor output cycles kaput; CYP11A1, cytochrome P450 family 11 subfamily A member 1; E2, estradiol; GnRH, gonadotropin‐releasing hormone; LH, luteinizing hormone; P4, progesterone; SCN, suprachiasmatic nucleus; STAR, steroidogenic acute regulatory protein; TTFL, transcription–translation feedback loop.

### 2.3 Circadian Remodeling in OC: Network Disruption, Driving Mechanisms, and Heterogeneity

OC development is accompanied by extensive circadian network remodeling characterized by reduced oscillation amplitude, phase shifts, and cellular desynchronization rather than complete clock loss [19]. TCGA/GEO‐based studies have identified associations between clock gene alterations, prognosis, and immune features; however, these relationships are strongly influenced by histological subtype, tumor purity, and microenvironmental composition, highlighting substantial heterogeneity [71]. For example, PER1 expression correlates with immune infiltration and clinical outcomes, suggesting that circadian dysregulation represents a context‐dependent network rather than universal clock suppression [72]. Through regulation of cell cycle, DNA damage responses, and metabolism, circadian disruption may contribute to tumor progression and therapeutic tolerance, although causal mechanisms require further validation [73, 74].

Epigenetic remodeling is a key mechanism underlying circadian alteration in OC. Yeh et al. [27] demonstrated that ARNTL (BMAL1) promoter methylation and H3K27me3 enrichment occur in a subset of OC cells, while EZH2 inhibition restores ARNTL expression, suppresses tumor growth, enhances cisplatin sensitivity, and reestablishes rhythmic c‐MYC activity. However, ARNTL/BMAL1 silencing is subtype‐ and context‐dependent, consistent with the heterogeneous methylation landscape of OC [75].

Additionally, MYC‐mediated repression of BMAL1/CLOCK/NPAS2 and HIF‐1α–BMAL1 interactions under hypoxia further reshape circadian networks and metabolic adaptation [76, 77].

Moreover, single‐cell and spatial studies reveal heterogeneous clock activity among tumor cells and microenvironmental components, emphasizing the need for cell‐specific and time‐resolved analyses [78]. Therefore, cell‐specific and time‐resolved approaches are required to define the functional consequences of circadian remodeling in OC.

## 3 DNAm‐Mediated Rewriting of the Circadian Clock

### 3.1 Promoter CpG Island Hypermethylation: “Source Silencing” of Core Clock Transcription

Promoter CpG island hypermethylation is a well‐established mechanism of long‐term transcriptional repression in cancer epigenetics [79]. Accumulation of 5‐methylcytosine (5mC) at promoter regions of core clock genes can impair transcriptional initiation and recruit methylation readers such as MBDs and MeCP2, promoting histone deacetylation, repressive chromatin formation, and persistent clock gene silencing. These processes contribute to reduced circadian amplitude and cellular desynchronization in tumors [48, 51, 79, 80, 81].

In OC, promoter methylation alterations are closely associated with tumor progression, prognosis, and therapeutic resistance. Genome‐wide methylation studies have identified extensive differences between cisplatin‐sensitive and resistant OC cells, with some alterations reversible after demethylating treatment, supporting a role for DNAm in regulating resistance‐associated transcriptional states [14]. Multi‐omics analyses further indicate that circadian gene dysregulation is associated with survival and immune characteristics, suggesting a potential link between methylation‐mediated clock regulation and therapy response [18]. PER2 represents a mechanistic example linking circadian regulation to platinum resistance. In cisplatin‐resistant OC cells, PER2 downregulation is accompanied by promoter CpG hypermethylation, whereas PER2 restoration enhances cisplatin sensitivity, suppresses PI3K/AKT signaling, and reduces MDR1/MRP1 expression, implicating PER2 in drug efflux, apoptosis, and EMT regulation [82]. However, DNAm alterations may reflect persistent resistant states rather than acute drug responses, as platinum resistance involves widespread CpG changes, whereas short‐term cisplatin exposure induces limited methylation and transcriptional alterations [83].

Notably, promoter hypermethylation does not universally silence clock genes, as its effects depend on methylation density, chromatin context, transcription factor accessibility, and additional epigenetic regulation [84]. Given the influence of tumor heterogeneity and cellular composition on methylation–expression relationships [85], the clinical significance of circadian gene methylation in OC requires further validation through subtype‐specific and longitudinal studies (Figure 2).

**FIGURE 2:**
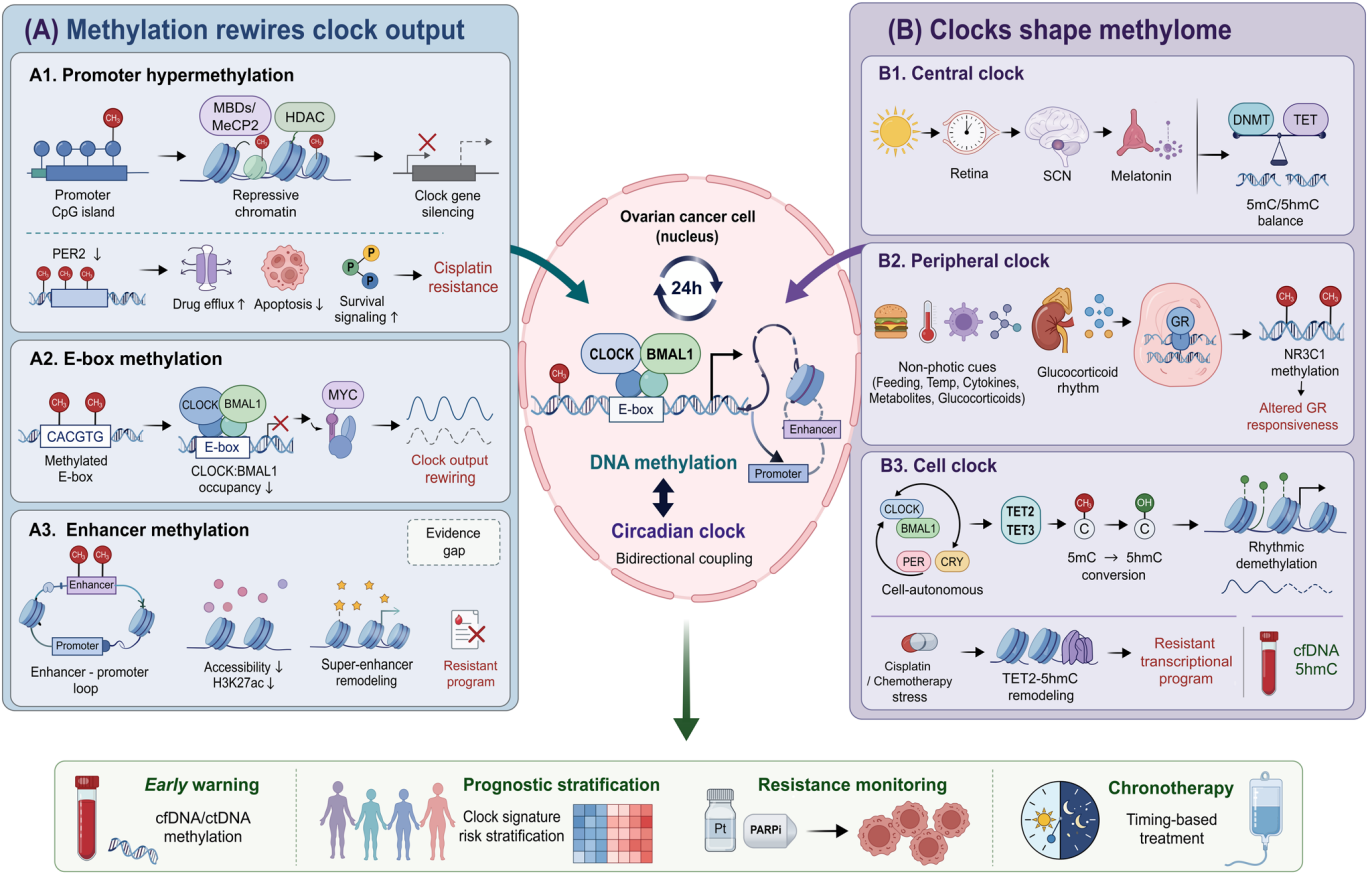
Bidirectional DNA methylation–circadian clock regulation in ovarian cancer progression and therapy response. (A) DNA methylation reshapes circadian clock outputs in ovarian cancer. Promoter, E‐box, and enhancer methylation alters CLOCK:BMAL1 transcriptional activity, induces clock gene silencing and chromatin remodeling, and contributes to resistant phenotypes through transcriptional rewiring. (B) Circadian clocks regulate DNA methylome dynamics. Central, peripheral, and cellular clocks influence DNMT/TET‐mediated methylation remodeling through hormonal and metabolic cues, while chemotherapy stress further alters epigenetic states. This bidirectional coupling provides potential biomarkers for risk stratification, resistance monitoring, and chronotherapy. 5hmC, 5‐hydroxymethylcytosine; 5mC, 5‐methylcytosine; cfDNA, circulating cell‐free DNA; CpG, cytosine–phosphate–guanine; ctDNA, circulating tumor DNA; DNMT, DNA methyltransferase; GR, glucocorticoid receptor; H3K27ac, histone H3 lysine 27 acetylation; HDAC, histone deacetylase; MBDs, methyl‐CpG‐binding domain proteins; MeCP2, methyl‐CpG‐binding protein 2; MYC, MYC proto‐oncogene; NR3C1, nuclear receptor subfamily 3 group C member 1; PARPi, poly(ADP‐ribose) polymerase inhibitor; TET, ten‐eleven translocation methylcytosine dioxygenase.

### 3.2 Cis‐Regulatory Element (E‐Box) Methylation: Loss of TF Occupancy and Binding Landscape Rewiring

CLOCK circadian transcription depends not only on core clock gene expression but also on phase‐specific occupancy of genome‐wide cis‐regulatory elements [34]. E‐box motifs (CANNTG, particularly CACGTG) serve as primary binding sites for CLOCK:BMAL1 and are widely distributed in promoter and enhancer regions. Under physiological conditions, CLOCK:BMAL1 recognizes these motifs within nucleosomal chromatin and promotes rhythmic transcription through coordinated interactions with chromatin components [34]. Structural studies further show that cooperative binding to paired E‐box elements enhances DNA accessibility and contributes to robust circadian oscillations [34, 86].

DNAm within E‐box motifs can alter transcription factor accessibility and binding affinity, thereby disrupting CLOCK transcriptional regulation [34, 50]. CACGTG‐containing regions occupied by BMAL1:CLOCK are generally hypomethylated, whereas methylation of critical cytosines reduces CLOCK binding, suppresses transcriptional activation, and weakens circadian amplitude [87, 88]. Moreover, altered E‐box methylation may reshape competition between CLOCK and other bHLH transcription factors, contributing to transcriptional rewiring [89].

In OC, circadian abnormalities associated with prognosis and immune features may reflect altered CLOCK regulatory activity rather than complete clock gene silencing [1, 18]. This mechanism may be particularly relevant in HGSOC, where MYC amplification or gain can enhance competition for E‐box‐containing regulatory regions [90]. MYC further suppresses circadian activity through REV‐ERB induction and MYC–MIZ1‐mediated repression of BMAL1 and CLOCK, weakening circadian oscillations and altering clock–cell‐cycle interactions [76]. Therefore, methylation changes at E‐box motifs and adjacent regulatory regions may function as context‐dependent modulators of transcription factor binding, driving circadian rewiring during OC progression or therapeutic stress and contributing to heterogeneous clinical and immune phenotypes [1, 18] (Figure 2).

### 3.3 Distal Enhancer Methylation: Topological Rewiring of Spatiotemporal Transcriptional Networks

Distal enhancers are essential cis‐regulatory elements that regulate gene transcription through long‐range enhancer–promoter interactions mediated by three‐dimensional chromatin organization and coordinated transcription factor recruitment [18, 91]. Genome‐wide studies have shown that CLOCK exhibits phase‐specific binding across enhancer regions, accompanied by rhythmic changes in chromatin accessibility, H3K27ac enrichment, and transcriptional activity, indicating that circadian outputs are largely governed by dynamic distal regulatory networks [31, 92].

DNAm at enhancers functions as a regulator of regulatory network architecture rather than a simple gene switch. Methylation changes at enhancer‐associated CpG regions can alter chromatin accessibility, transcription factor binding, cofactor recruitment, and enhancer–promoter communication through regulators such as CTCF [93, 94]. Temporal methylation patterns correlate with chromatin accessibility dynamics, and rhythmic demethylation of specific CpG regions can cooperate with enhancers to regulate clock‐controlled gene expression, highlighting the role of DNA modifications in circadian enhancer regulation [28, 95, 96, 97, 98].

In OC, distal enhancer remodeling contributes to adaptive malignant states. Shang et al. demonstrated that cisplatin exposure induces new distal enhancers and super‐enhancer landscapes in platinum‐resistant OC models, sustaining resistance‐associated transcriptional programs [99]. Therapy‐induced enhancer networks may further promote stemness and resistance phenotypes, while HGSOC methylome studies indicate that regional methylation patterns are associated with chemotherapy tolerance and poor outcomes [14, 100].

Because enhancers encode temporal regulatory information, their remodeling may influence circadian outputs by altering CLOCK recruitment and regulatory connectivity without complete clock gene silencing [92, 101]. Therefore, enhancer remodeling in OC may alter CLOCK:BMAL1 recruitment and circadian output connectivity without requiring complete silencing of core clock genes [99].

However, whether resistance‐associated enhancers exhibit circadian oscillation or methylation‐dependent regulation remains unclear. Integrated analyses of enhancer landscapes, circadian profiling, and methylomes are required to define this potential regulatory axis (Figure 2).

## 4 How the Circadian Clock Shapes the DNAm Landscape

### 4.1 The Central Clock as a System‐Level Epigenetic Memory

The mammalian circadian system is hierarchically organized, with the suprachiasmatic nucleus (SCN) acting as the central pacemaker that synchronizes peripheral clocks through systemic outputs rather than directly regulating tumor cells [102]. In OC, the central clock shapes the temporal environment of peripheral tissues and the tumor microenvironment through regulation of sleep–wake rhythms, body temperature, behavioral activity, and melatonin secretion [19].

Night‐shift work and nocturnal light exposure are major disruptors of circadian entrainment. Although Bhatti et al. [70, 103] reported an association between night‐shift work and increased risk of aggressive epithelial OC and borderline ovarian tumors, subsequent meta‐analyses showed no consistent overall association, likely due to differences in exposure assessment, shift duration, chronotype, subtype, and confounding factors. Melatonin represents a key interface between light signaling and tumor biology. Circadian disruption reduces nocturnal melatonin secretion, and lower melatonin levels have been observed in OC patients [104, 105, 106]. Experimental studies indicate that melatonin may inhibit OC progression through regulation of proliferation, apoptosis, oxidative stress, cell‐cycle control, and immune responses [107, 108, 109, 110]. It may also influence DNAm through DNMT/TET pathways and 5mC/5hmC regulation [111, 112]. However, a direct causal link between central circadian disruption, melatonin‐mediated epigenetic remodeling, and OC progression remains unproven. Integrated studies combining circadian exposure assessment, melatonin profiling, and multi‐omics analyses are required to clarify this potential mechanism (Figure 2).

### 4.2 Peripheral Clocks Translate Non‐Photonic Cues Into Tissue‐Specific Methylation Baselines

Peripheral clocks are continuously reset by non‐photic zeitgebers, including feeding patterns, metabolic signals, inflammation, temperature changes, and glucocorticoids [113, 114]. Glucocorticoid signaling represents a major peripheral entrainment pathway, rapidly shifting clock gene expression in peripheral tissues while exerting limited effects on SCN rhythms [115]. In humans, hydrocortisone administration alters PER2, PER3, and BMAL1 expression phases in PBMCs without substantially affecting central circadian markers [116]. Beyond phase resetting, these signals influence metabolic and stress environments and may shape epigenetic states, with DNAm potentially serving as a molecular record of temporal and physiological conditions [43, 117].

In OC, glucocorticoid‐mediated circadian regulation may depend on the expression and chromatin accessibility of the glucocorticoid receptor (GR), encoded by NR3C1. Aberrant NR3C1 promoter methylation has been identified in OC, and independent cohorts have linked NR3C1 methylation with cortisol‐related phenotypes [9, 118, 119, 120]. Thus, NR3C1 methylation may function as an epigenetic threshold regulating glucocorticoid responsiveness and contribute to interindividual differences in circadian adaptation during tumor progression and treatment. Further integration of glucocorticoid exposure, NR3C1 methylation, circadian profiles, and clinical outcomes is required to define this regulatory interface (Figure 2).

### 4.3 Cellular Clocks Gate Demethylation Capacity and Generate Locus‐Specific Rhythmic Demethylation Units

Cellular clocks are autonomous circadian oscillators driven by the CLOCK/BMAL1–PER/CRY transcription–TTFL, generating intrinsic temporal organization independent of systemic rhythms [121, 122]. Beyond controlling clock gene expression, cellular clocks regulate transcriptional programs, chromatin states, and stress responses, thereby shaping cellular adaptability [123, 124, 125, 126].

Rhythmic DNAm remodeling represents an important layer of circadian regulation. Clock components can modulate active demethylation pathways involving TET enzymes and 5‐hydroxymethylcytosine (5hmC), creating temporal windows for regulatory DNA demethylation [28, 125, 127]. Bmal1 regulates stress‐associated epigenetic responses through Tet3 and 5hmC dynamics, while circadian demethylation of specific CpG regions cooperates with rhythmic enhancers to regulate clock‐controlled transcription [28, 125].

In OC, the TET2–5hmC axis may be remodeled during therapeutic stress and resistance evolution, contributing to adaptive transcriptional states [128, 129, 130]. Serum cfDNA 5hmC profiling also shows potential for predicting chemotherapy resistance in HGSOC [130]. Thus, clock–DNAm coupling may influence OC progression and treatment response, requiring validation through time‐resolved epigenomics, single‐cell analyses, and functional studies (Figure 2).

## 5 Circadian Clock Genes and Their Methylation Patterns as Biomarkers

### 5.1 Core Clock Gene‐Specific Hypermethylation as an Early Warning Signal

OC, particularly HGSOC, remains highly lethal due to the lack of sufficiently sensitive and specific early detection strategies [1]. CA125 has limited performance in early‐stage disease and may be elevated in benign conditions, highlighting the need for additional molecular biomarkers [1]. DNAm alterations have emerged as promising molecular indicators because of their stability, detectability, and compatibility with liquid biopsy approaches using ctDNA and cfDNA [15].

Within the circadian–OC regulatory axis, methylation‐mediated silencing of core clock genes represents a potential biomarker source. Yeh et al. [27] showed that ARNTL (BMAL1) promoter methylation occurs in a subset of OC cells and is associated with repressive chromatin states, reduced expression, impaired cisplatin sensitivity, and altered c‐MYC rhythmicity. ctDNA/cfDNA methylation profiling shows early detection potential in OC, with marker selection and pre‐analytics affecting performance; multimarker strategies may improve accuracy [15, 131].

However, bulk methylation profiles may be confounded by tumor purity and cellular heterogeneity. Single‐cell multi‐omics studies in HGSOC have revealed distinct methylation patterns among tumor lineages, indicating that bulk analyses may overlook rare subpopulations with metastatic potential [132]. Clinical application further requires standardized methylation thresholds, as ARNTL/BMAL1 hypermethylation varies according to CpG sites, sample type, detection platform, tumor DNA fraction, and analytical methods [133]. For cfDNA/ctDNA panels, integrated scoring models and standardized pre‐analytical procedures may be required.

Therefore, circadian‐related methylation loci should currently be considered complementary candidate markers rather than independent biomarkers. Their diagnostic value requires validation across early‐stage OC, high‐risk populations, and diverse subtypes, with assessment beyond CA125 and established methylation panels [15, 131]. (Table 1).

**TABLE 1:** Potential clinical applications, analytical considerations, and current limitations of circadian‐related DNA methylation biomarkers in ovarian cancer.

Biomarker or molecular feature	Potential application	Evidence and principal findings	Heterogeneity and analytical considerations	Threshold or scoring strategy	Evidence level and key limitations	References
ARNTL/BMAL1 promoter methylation	Early detection; molecular stratification; platinum‐response prediction	Detected in a subset of OC cells and associated with ARNTL silencing, altered c‐MYC rhythms, and cisplatin response	Varies by subtype, CpG site, tumor purity, sample type, and assay platform; bulk analysis may mask minor subclones	No universal cutoff; locus‐ and assay‐specific thresholds or composite scores are needed	Preclinical evidence; limited clinical and early‐stage validation; added value beyond CA125 is unknown	[27, 133]
PER2 promoter methylation	Platinum‐resistance monitoring; response stratification characteristics and outcomes; circadian signatures are associated with prognosis and immune features.	Associated with reduced PER2, PI3K/AKT activation, EMT, apoptosis inhibition, and drug efflux in resistant OC models	Mainly based on SKOV3 models; applicability across HGSOC and tissue–plasma concordance remain unclear	No validated resistance cutoff; change from pretreatment baseline may be more informative	Preclinical evidence; requires longitudinal plasma validation; causal relevance remains uncertain	[32, 82]
Global or regional HGSOC methylation subtypes	Prognostic stratification; recurrence‐risk assessment	Methylation‐defined subgroups show different survival, residual disease, and molecular features	Influenced by tumor purity, stromal/immune admixture, cell of origin, platform, and clinical covariates	Based mainly on clustering, classifier probabilities, or multigene scores	Retrospective clinical evidence; requires prospective validation against FIGO stage, residual disease, and BRCA/HRD status	[134, 135, 136, 137, 138]
OriPrint cell‐of‐origin signature	Biological subclassification; prognosis	Distinguishes fallopian tube epithelial‐like and ovarian surface epithelial‐like HGSOC subgroups	Influenced by tumor purity, lineage admixture, reference cells, platform, and cohort composition	Classifier probability or composite score; no universal cutoff	Retrospective evidence; requires independent validation and assessment of added prognostic value	[139]
Circadian‐related molecular signatures	Prognostic and immune stratification	Circadian gene signatures correlate with survival and immune characteristics	Derived mainly from bulk transcriptomes and affected by cellular composition, sampling time, and phase desynchronization	Cohort‐specific risk scores; integrated methylation–expression models are needed	Retrospective bioinformatic evidence; indirect support for DNAm–clock coupling; no prospective validation	[140, 141]
cfDNA/ctDNA methylation panels incorporating circadian loci	Early detection; recurrence and resistance monitoring	Multilocus methylation panels may improve OC detection, especially when combined with mutations or protein markers	Affected by low tumor fraction, hematopoietic cfDNA, sample handling, bisulfite loss, and sequencing depth	Multilocus scores are preferable; cutoffs should be trained and independently validated	Early translational evidence; circadian loci remain unvalidated; stage‐specific performance and comparison with CA125 are needed	[15, 131]
Longitudinal clock‐related ctDNA methylation changes	Dynamic response and resistance monitoring	Serial ctDNA changes may precede imaging or CA125 progression in some settings	Changes may reflect tumor burden, DNA shedding, treatment effects, clonal selection, and sampling interval	Patient‐specific baseline changes or minimal detectable change may be preferable	Early translational evidence; no validated clock‐specific algorithm; prospective outcome correlation is needed	[3, 142, 143, 144]

Abbreviations: AKT, protein kinase B; ARNTL, aryl hydrocarbon receptor nuclear translocator‐like; BMAL1, brain and muscle ARNT‐like 1; BRCA, BReast CAncer susceptibility gene; CA125, cancer antigen 125; cfDNA, cell‐free DNA; c‐MYC, cellular MYC proto‐oncogene; CpG, cytosine‐phosphate‐guanine; ctDNA, circulating tumor DNA; DNAm, DNA methylation; EMT, epithelial–mesenchymal transition; FIGO, International Federation of Gynecology and Obstetrics; HGSOC, high‐grade serous ovarian cancer; HRD, homologous recombination deficiency; OC, ovarian cancer; PER2, period circadian regulator 2; PI3K, phosphoinositide 3‐kinase; SKOV3, Sloan‐Kettering ovarian cancer cell line 3.

### 5.2 Rhythmic Epigenomic Features and Prognostic Stratification

HGSOC exhibits substantial clinical heterogeneity, with patients of similar FIGO stages and treatment regimens showing different recurrence risks and survival outcomes, indicating that conventional parameters cannot fully reflect tumor intrinsic states [145]. DNAm‐based epigenomic patterns provide stable stratification features, and integration with circadian activity and immune characteristics may add a temporal dimension to prognostic classification.

Global methylation profiles have prognostic relevance in HGSOC. Lo Riso et al. [139] developed the OriPrint DNAm‐based cell‐of‐origin classifier and identified fallopian tube epithelial‐like and ovarian surface epithelial‐like subgroups, with the latter associated with more aggressive phenotypes and poorer outcomes. Zhao et al. [140] identified circadian‐related genes linked to prognosis and immune features using TCGA OC data, while Xing et al. [141] showed that TIMELESS promotes OC progression and correlates with macrophage infiltration. However, methylation‐based prognostic interpretation remains context‐dependent. Wang et al. [134] identified four HGSOC methylation subtypes with distinct outcomes, emphasizing the importance of broader epigenomic backgrounds. Therefore, circadian‐related methylation features should serve as complementary components of integrated prognostic models.

Therefore, circadian‐related methylation features should be incorporated into integrated prognostic models with standardized validation and adjustment for clinical factors, BRCA/HRD status, tumor purity, and sampling time [134, 135, 136, 137].

Overall, circadian‐related methylation features represent complementary prognostic dimensions that require further validation to determine their incremental value beyond established clinical and molecular models. (Table 1).

### 5.3 Epigenetic Consolidation of Clock Genes for Dynamic Monitoring of Platinum Resistance

Platinum‐based chemotherapy combined with taxanes remains the standard first‐line treatment for HGSOC; however, most patients eventually relapse and develop platinum resistance after initial responses [4, 138]. Resistance reflects continuous clonal evolution under therapeutic pressure, making early detection of resistant states before radiographic or CA125 changes clinically important [3, 5]. ctDNA enables serial monitoring of heterogeneous tumor‐derived genetic and epigenetic alterations and has shown potential for assessing treatment response and detecting progression earlier than conventional approaches in some settings [3, 142, 143]. Longitudinal TP53‐mutant ctDNA dynamics and personalized ctDNA strategies further support its utility, although interpretation requires consideration of tumor burden, lesion distribution, and treatment‐induced cell death [144].

Circadian dysregulation may contribute to resistance evolution when stabilized by epigenetic remodeling. Wang et al. [82] demonstrated that PER2 loss in cisplatin‐resistant OC models activates PI3K/AKT signaling, promotes EMT‐like changes, suppresses apoptosis, and enhances drug efflux, with reduced PER2 expression accompanied by promoter CpG methylation, suggesting a potential resistance‐associated marker. Evidence from SKOV3 models remains insufficient to confirm whether PER2 methylation reliably reflects resistant clone emergence in HGSOC plasma. Other clock regulators, including CLOCK, have also been linked to reduced cisplatin sensitivity through regulation of cell‐cycle and stress‐response pathways [146]. However, whether these methylation alterations directly drive resistance or reflect treatment‐selected states remains unclear [32].

Future longitudinal studies integrating clock‐related methylation, ctDNA alterations, and clinical parameters are required to determine their predictive value for resistance evolution and treatment adaptation. (Table 1).

## 6 Therapeutic Implications: From Epigenetic Reprogramming to Chronotherapy‐Based Precision Treatment

### 6.1 Epigenetic Reprogramming as a Sensitization Hub

Platinum resistance is frequently associated with reversible epigenetic reprogramming, including abnormal DNAm and repressive chromatin remodeling, which stabilize resistance‐related transcriptional states involving stress adaptation, apoptosis, immune regulation, and differentiation [147, 148]. Thus, the therapeutic value of epigenetic resetting lies in reversing these constraints and restoring drug sensitivity through DNA demethylation or chromatin relaxation [6, 149].

In OC, DNA demethylating agents have emerged as potential sensitizers. A randomized Phase II trial showed that guadecitabine combined with carboplatin could support the strategy of epigenetic priming followed by platinum rechallenge in platinum‐resistant recurrent OC [150], with earlier studies demonstrating feasibility [151]. Moreover, combinations of guadecitabine or azacitidine with immune checkpoint inhibitors suggest that epigenetic modulation can reshape the tumor microenvironment and enhance anti‐tumor immunity [149, 152]. HDAC inhibition with vorinostat has also shown potential for improving chemotherapy synergy [153]. Collectively, epigenetic resetting represents a promising strategy to overcome resistance‐associated states and optimize combination therapies in OC (Table 2).

**TABLE 2:** Therapeutic implications of epigenetic reprogramming and chronotherapy in ovarian cancer.

Therapeutic strategy/molecular target	Mechanistic rationale and key findings	Therapeutic implication	Current limitations and future directions	References
Epigenetic reprogramming with DNA demethylating agents	Abnormal DNA methylation contributes to platinum‐resistant transcriptional states; guadecitabine‐mediated DNA demethylation may restore platinum sensitivity and enhance response to platinum rechallenge	Epigenetic priming followed by chemotherapy may overcome resistant phenotypes in recurrent OC	Clinical benefit remains heterogeneous; optimal patient selection biomarkers and methylation‐based response predictors require validation	[147, 148, 149, 150, 151]
DNA methylation modulation combined with immunotherapy	DNMT inhibition can reshape tumor immune microenvironment and enhance anti‐tumor immune responses when combined with immune checkpoint blockade	Potential strategy to improve immunotherapy responsiveness in OC	Need identification of immune–epigenetic biomarkers and confirmation in larger clinical trials	[149, 152]
HDAC inhibition and chromatin remodeling strategies	HDAC inhibitors such as vorinostat can induce chromatin relaxation and enhance chemotherapy‐induced tumor cell killing	Combination therapy approach for overcoming chemoresistance	Limited clinical evidence; optimal combinations, dosing schedules, and toxicity profiles remain unclear	[153]
Chronotherapy‐based chemotherapy scheduling	Circadian rhythms influence drug metabolism, DNA repair, hematopoiesis, and normal tissue tolerance; timed chemotherapy may optimize efficacy and reduce toxicity	Potential approach for improving treatment tolerability and therapeutic index	Early studies mainly demonstrated toxicity reduction rather than improved tumor control; modern molecular stratification is lacking	[154, 155, 156, 157, 158]
Circadian regulation of DNA repair and platinum/PARP inhibitor response	CRY1 regulates circadian oscillations of DNA end resection and homologous recombination repair, suggesting time‐dependent variation in DNA repair capacity	Provides mechanistic basis for treatment‐time optimization of platinum agents and PARP inhibitors	Clinical relevance of repair oscillations remains unconfirmed; requires integration with pharmacokinetics, pharmacodynamics, and molecular biomarkers	[159]
BRCA1 promoter methylation as a theragnostic biomarker	BRCA1 promoter methylation is associated with homologous recombination deficiency and improved sensitivity to platinum chemotherapy and PARP inhibitors; complete allele‐specific methylation status provides greater predictive accuracy	Potential biomarker for selecting patients likely to benefit from HR‐targeted therapies	Binary methylation classification may be insufficient; allele‐specific methylation assessment and standardized detection methods are needed	[160, 161]
Dynamic HR pathway methylation monitoring during treatment evolution	Chemotherapy‐induced BRCA1 demethylation can restore BRCA1 expression and promote platinum resistance; RAD51C demethylation contributes to PARP inhibitor resistance	Longitudinal methylation monitoring may predict acquired resistance and guide treatment adaptation	Requires integration of ctDNA, clonal evolution, treatment pressure, and temporal sampling strategies	[162, 163, 164]
Integration of circadian regulation with HR methylation status	Circadian control of HR activity and diurnal BRCA1/BRCA2 expression patterns suggest that DNA repair methylation states may interact with temporal regulation	Conceptual framework for defining therapeutic windows in platinum‐ and PARP inhibitor‐based therapies	Direct evidence linking clock‐controlled methylation states with treatment timing is currently insufficient	[159, 165]

Abbreviations: BRCA1, breast cancer gene 1; BRCA2, breast cancer gene 2; CRY1, cryptochrome circadian regulator 1; DNMT, DNA methyltransferase; HDAC, histone deacetylase; HR, homologous recombination; HRD, homologous recombination deficiency; KOMET, the Korea PARP inhibitor biomarker study; PARP, poly(ADP‐ribose) polymerase; PD, pharmacodynamic; PK, pharmacokinetic; RAD51C, RAD51 paralog C.

### 6.2 Chronotherapy as Time‐Dimensioned Precision Treatment

Therapeutic responses in OC may be influenced not only by drug selection and dosage but also by host circadian states. Chronotherapy incorporates treatment timing into therapeutic strategies by exploiting circadian variations in drug metabolism, tissue tolerance, hematopoiesis, and DNA damage repair to optimize efficacy and reduce toxicity [154, 155, 156].

Early clinical studies suggested potential treatment‐time effects in OC. Hrushesky et al. [157] reported that morning Adriamycin followed by evening cisplatin administration improved tolerability in advanced OC, while Lévi et al. [158] demonstrated reduced hematological and renal toxicity with circadian scheduling of anthracycline/cisplatin therapy. However, these studies were limited by small sample sizes, outdated regimens, and lack of molecular stratification, with benefits mainly observed in toxicity reduction rather than tumor control.

Recent studies provide mechanistic support for OC chronotherapy. Romero‐Franco et al. [159] demonstrated circadian regulation of DNA end resection and homologous recombination (HR) repair by CRY1, suggesting that temporal DNA repair capacity may influence responses to platinum agents and PARP inhibitors. Malhan et al. [22] further linked PARP inhibitor‐associated circadian disruption with treatment toxicity, although causality remains unclear. Therefore, current evidence supports biological plausibility but not routine clinical application.

Future randomized trials should integrate treatment timing, molecular biomarkers, pharmacokinetic/pharmacodynamic parameters, and liquid biopsy monitoring to determine the clinical value of chronotherapy in precision OC management (Table 2).

### 6.3 Therapeutic Time Windows and Efficacy Evidence of DNAm–Clock Coupling

In OC, the most clinically validated association between DNAm and therapeutic response involves the HR repair pathway. The KOMET study demonstrated that BRCA1 promoter methylation was associated with improved responses to PARP inhibitors and platinum chemotherapy, supporting its potential as a theragnostic biomarker [160]. Further analyses indicated that predictive accuracy depends on complete BRCA1 allele methylation status rather than binary methylation classification alone [161]. Importantly, HR‐related methylation states are dynamic. Menghi et al. [162, 163] showed that chemotherapy‐induced BRCA1 promoter demethylation restored BRCA1 expression and contributed to platinum resistance, while RAD51C promoter demethylation was associated with PARP inhibitor resistance.

The convergence between HR methylation and circadian regulation lies in their shared influence on DNA repair capacity. Romero‐Franco et al. [159] demonstrated circadian oscillations of DNA end resection and HR repair regulated by CRY1, suggesting that temporal variation in repair activity may affect responses to platinum agents and PARP inhibitors. Consistently, diurnal BRCA1/BRCA2 expression patterns provide further support for integrating temporal regulation into HR‐targeted therapies [165]. However, treatment‐specific evolutionary trajectories should be considered, as RAD51C demethylation after niraparib resistance may restore PARP inhibitor resistance while preserving carboplatin sensitivity [164].

Therefore, future models should integrate HR methylation profiles, allele‐specific status, ctDNA dynamics, clonal evolution, treatment pressure, and circadian variables to better define therapeutic windows. (Table 2).

## 7 Conclusions and Perspectives

The initiation, progression, and therapeutic resistance of OC arise from dynamic interactions among tumor states, microenvironmental pressures, treatment selection, and temporal regulation rather than isolated genetic events [11, 163, 166]. DNAm, as a stable yet plastic epigenetic layer, reflects tumor evolution and regulates circadian outputs through promoters, E‐box elements, enhancers, and chromatin organization [14, 124, 167]. Conversely, circadian networks influence therapeutic responses by modulating DNA repair, metabolism, cell‐cycle progression, immunity, and drug tolerance [22, 140]. Thus, DNAm–circadian integration provides a temporal framework for understanding OC adaptation. Mechanistically, promoter CpG hypermethylation may silence core clock genes, while methylation remodeling at E‐box motifs and enhancers can alter CLOCK binding, chromatin accessibility, and transcriptional programs [27, 87, 168, 169]. Meanwhile, circadian signals such as melatonin, glucocorticoids, TET/5hmC pathways, and enhancer‐associated demethylation can reshape methylation landscapes [111, 170, 171, 172], supporting a bidirectional regulatory network. Circadian‐related methylation signatures may offer complementary value for early detection, prognosis, resistance monitoring, and therapy optimization. However, markers such as ARNTL/BMAL1 methylation remain exploratory, and their clinical utility requires validation beyond current CA125 and methylation‐based panels [15, 16, 17, 27]. Integrating HR methylation profiles, ctDNA dynamics, and circadian variables may improve prediction of PARP inhibitor and platinum responses [165].

Future studies should combine time‐resolved multi‐omics, single‐cell/spatial epigenomics, functional validation, and chronotherapy trials. Overall, DNAm–circadian coupling represents a context‐dependent regulatory framework that may enable dynamic monitoring and precision therapy in OC.

## Author Contributions

**Min Xing:** writing – original draft, resources. **Chang Zhou:** resources, writing – review and editing. **Yi Tang:** writing – review and editing. **Shuhui Xu:** writing – review and editing. **Shasha Zhou:** writing – review and editing. **Weiai Liu:** writing – review and editing. **Huiping Liu:** conceptualization, supervision, writing – review and editing.

## Funding

This work was supported by the Hunan Provincial Natural Science Foundation, Joint Project (China, Grant No. 2025JJ90029), the Hunan University of Chinese Medicine, Intellectual Property Transformation and Application Special Fund (China, Grant No. CGZH08), the Hunan Provincial Department of Science and Technology, Key R&D Program (China, Grant No. 2025JK2111, Weiai Liu), the Hunan University of Chinese Medicine, Graduate Innovation Project (China, Grant No. CX20251185, Min Xing), and the Open Fund of the Provincial‐Ministerial Co‐constructed National Key Laboratory of Chinese Herbal Powder and Innovative Drugs (China, Grant No. 25PTKF1011, Shasha Zhou).

## Conflicts of Interest

The authors declare no conflicts of interest.

## References

[1] B. Narayanan , T. Buddenkotte , H. Smith , et al., “Growth Kinetics of High‐Grade Serous Ovarian Cancer: Implications for Early Detection,” British Journal of Cancer 133, no. 4 (2025): 533–538, 10.1038/s41416-025-03082-6. PMC12356848 40506518

[2] M. K. Hong and D. C. Ding , “Early Diagnosis of Ovarian Cancer: A Comprehensive Review of the Advances, Challenges, and Future Directions,” Diagnostics (Basel) 15, no. 4 (2025): 406, 10.3390/diagnostics15040406. PMC11854769 40002556

[3] E. Rios‐Doria , J. B. Reichel , M. R. Radke , et al., “Dynamic Monitoring of Recurrent Ovarian Cancer Using Serial ctDNA: A Real‐World Case Series,” Current Oncology 32, no. 10 (2025): 585, 10.3390/curroncol32100585. PMC12563156 41149505

[4] H. Li , J. J. Sheng , S. A. Zheng , et al., “Platinum‐Resistant Ovarian Cancer: From Mechanisms to Treatment Strategies,” Genes and Diseases 13, no. 2 (2025): 101801, 10.1016/j.gendis.2025.101801. PMC12688694 41376855

[5] M. J. Williams , I. Vázquez‐García , G. Tam , et al., “Tracking Clonal Evolution During Treatment in Ovarian Cancer Using Cell‐Free DNA,” Nature 647, no. 8090 (2025): 757–765, 10.1038/s41586-025-09580-0. PMC12629990 41034582

[6] N. Dedes , M. Liontos , D. Haidopoulos , et al., “Epigenetics in Ovarian Cancer: A Review of Current Knowledge and Future Perspectives,” Biomedicine 13, no. 11 (2025): 2820, 10.3390/biomedicines13112820. PMC12649953 41301911

[7] C. Herzog , A. Jones , I. Evans , et al., “Plasma Cell‐Free DNA Methylation Analysis for Ovarian Cancer Detection: Analysis of Samples From a Case‐Control Study and an Ovarian Cancer Screening Trial,” International Journal of Cancer 154, no. 4 (2024): 679–691, 10.1002/ijc.34757. PMC7617350 37861205

[8] G. Li , Y. Zhang , K. Li , et al., “Transformer‐Based AI Technology Improves Early Ovarian Cancer Diagnosis Using cfDNA Methylation Markers,” Cell Reports Medicine 5, no. 8 (2024): 101666, 10.1016/j.xcrm.2024.101666. PMC11384945 39094578

[9] X. M. Guo , H. Miller , K. Matsuo , L. D. Roman , and B. Salhia , “Circulating Cell‐Free DNA Methylation Profiles in the Early Detection of Ovarian Cancer: A Scoping Review of the Literature,” Cancers (Basel) 13, no. 4 (2021): 838, 10.3390/cancers13040838. PMC7923044 33671298

[10] T. Xie , K. A. Dickson , C. Yee , et al., “Targeting Homologous Recombination Deficiency in Ovarian Cancer With PARP Inhibitors: Synthetic Lethal Strategies That Impact Overall Survival,” Cancers 14, no. 19 (2022): 4621, 10.3390/cancers14194621. PMC9563432 36230543

[11] L. Y. Feng , B. B. Yan , Y. Z. Huang , and L. Li , “Abnormal Methylation Characteristics Predict Chemoresistance and Poor Prognosis in Advanced High‐Grade Serous Ovarian Cancer,” Clinical Epigenetics 13, no. 1 (2021): 141, 10.1186/s13148-021-01133-2. PMC8296752 34289901

[12] P. Gautam , S. Gupta , and M. Sachan , “Comprehensive DNA Methylation Profiling by MeDIP‐NGS Identifies Potential Genes and Pathways for Epithelial Ovarian Cancer,” Journal of Ovarian Research 17, no. 1 (2024): 83, 10.1186/s13048-024-01395-3. PMC11022481 38627856

[13] H. Cardenas , F. Fang , G. Jiang , et al., “Methylomic Signatures of High Grade Serous Ovarian Cancer,” Epigenetics 16, no. 11 (2021): 1201–1216, 10.1080/15592294.2020.1853402. PMC8813084 33289590

[14] D. W. Chan , W. Y. Lam , F. Chen , et al., “Genome‐Wide DNA Methylome Analysis Identifies Methylation Signatures Associated With Survival and Drug Resistance of Ovarian Cancers,” Clinical Epigenetics 13, no. 1 (2021): 142, 10.1186/s13148-021-01130-5. PMC8296615 34294135

[15] S. K. Terp , M. P. Stoico , K. Dybkær , and I. S. Pedersen , “Early Diagnosis of Ovarian Cancer Based on Methylation Profiles in Peripheral Blood Cell‐Free DNA: A Systematic Review,” Clinical Epigenetics 15, no. 1 (2023): 24, 10.1186/s13148-023-01440-w. PMC9926627 36788585

[16] L. Liang , Y. Zhang , C. Li , et al., “Plasma cfDNA Methylation Markers for the Detection and Prognosis of Ovarian Cancer,” eBioMedicine 83 (2022): 104222, 10.1016/j.ebiom.2022.104222. PMC9396542 35973389

[17] D. N. Buckley , J. P. Lewinger , G. Gooden , et al., “OvaPrint‐A Cell‐Free DNA Methylation Liquid Biopsy for the Risk Assessment of High‐Grade Serous Ovarian Cancer,” Clinical Cancer Research 29, no. 24 (2023): 5196–5206, 10.1158/1078-0432.CCR-23-1197. PMC10722131 37812492

[18] J. Zhu , Q. Zhou , M. Pan , and C. Zhou , “Multi‐Omics Analysis of the Prognosis and Therapeutic Significance of Circadian Clock in Ovarian Cancer,” Gene 788 (2021): 145644, 10.1016/j.gene.2021.145644. 33848579

[19] Y. Lee , “Roles of Circadian Clocks in Cancer Pathogenesis and Treatment,” Experimental and Molecular Medicine 53, no. 10 (2021): 1529–1538, 10.1038/s12276-021-00681-0. PMC8568965 34615982

[20] Y. Zhang , J. Lin , N. Liu , et al., “ARNTL Deregulation Promotes Ovarian Cancer Progression and Metastasis by Activating Cancer‐Associated Fibroblasts,” Journal of Ovarian Research 19, no. 1 (2026): 130, 10.1186/s13048-026-02044-7. PMC13041274 41742264

[21] F. S. Pektopal , S. Kum Özşengezer , and Z. S. Altun , “Chemoresistance in Ovarian Cancer and Association With Circadian Rhythm,” Reproductive Sciences 33 (2026): 1079–1090, 10.1007/s43032-026-02093-9. PMC13328239 42082821

[22] D. Malhan , J. Hesse , N. Nelson , et al., “Circadian Rhythm Disruption by PARP Inhibitors Correlates With Treatment Toxicity in Patients With Ovarian Cancer and Is a Predictor of Side Effects,” eBioMedicine 117 (2025): 105764, 10.1016/j.ebiom.2025.105764. PMC12173083 40382284

[23] M. Surti , A. Gupta , K. Janiyani , M. Adnan , and M. Patel , “The Role of Circadian Disruption, Sleep Dysregulation, Lifestyle Factors, and Metabolic Reprogramming in the Pathogenesis and Progression of Ovarian Cancer,” Journal of the National Cancer Center 6, no. 2 (2026): 189–203, 10.1016/j.jncc.2026.01.009. PMC13083629 42007213

[24] A. J. Ämmälä , T. P. M. Hancox , F. Qiuyu , et al., “Daily Rhythm in DNA Methylation and the Effect of Total Sleep Deprivation,” Journal of Sleep Research 34, no. 4 (2025): e14438, 10.1111/jsr.14438. PMC12215246 39675927

[25] G. Coppens , A. B. G. Cramer , I. Vanhorebeek , et al., “Aberrant DNA Methylation Within Circadian Entrainment and Clock Genes Two Years After Pediatric Critical Illness Is Associated With Impaired Physical and Neurocognitive Development,” Clinical Epigenetics 18, no. 1 (2025): 3, 10.1186/s13148-025-02027-3. PMC12763905 41276843

[26] S. Chen , W. Zhang , X. Li , Z. Cao , and C. Liu , “DNA Polymerase Beta Connects Tumorigenicity With the Circadian Clock in Liver Cancer Through the Epigenetic Demethylation of Per1,” Cell Death and Disease 15, no. 1 (2024): 78, 10.1038/s41419-024-06462-7. PMC10799862 38245510

[27] C. M. Yeh , J. Shay , T. C. Zeng , et al., “Epigenetic Silencing of ARNTL, a Circadian Gene and Potential Tumor Suppressor in Ovarian Cancer,” International Journal of Oncology 45, no. 5 (2014): 2101–2107, 10.3892/ijo.2014.2627. 25175925

[28] N. Misra , M. Damara , T. Ye , and P. Chambon , “The Circadian Demethylation of a Unique Intronic deoxymethylCpG‐Rich Island Boosts the Transcription of Its Cognate Circadian Clock Output Gene,” Proceedings of the National Academy of Sciences of the United States of America 120, no. 8 (2023): e2214062120, 10.1073/pnas.2214062120. PMC9974474 36791105

[29] J. S. Takahashi , “Transcriptional Architecture of the Mammalian Circadian Clock,” Nature Reviews. Genetics 18, no. 3 (2017): 164–179, 10.1038/nrg.2016.150. PMC5501165 27990019

[30] M. Del Olmo , S. Legewie , M. Brunner , et al., “Network Switches and Their Role in Circadian Clocks,” Journal of Biological Chemistry 300, no. 5 (2024): 107220, 10.1016/j.jbc.2024.107220. PMC11044057 38522517

[31] M. Qu , H. Qu , Z. Jia , and S. A. Kay , “HNF4A Defines Tissue‐Specific Circadian Rhythms by Beaconing BMAL1::CLOCK Chromatin Binding and Shaping the Rhythmic Chromatin Landscape,” Nature Communications 12, no. 1 (2021): 6350, 10.1038/s41467-021-26567-3. PMC8566521 34732735

[32] F. Fagiani , D. Di Marino , A. Romagnoli , et al., “Molecular Regulations of Circadian Rhythm and Implications for Physiology and Diseases,” Signal Transduction and Targeted Therapy 7, no. 1 (2022): 41, 10.1038/s41392-022-00899-y. PMC8825842 35136018

[33] Y. O. Abe , H. Yoshitane , D. W. Kim , et al., “Rhythmic Transcription of Bmal1 Stabilizes the Circadian Timekeeping System in Mammals,” Nature Communications 13, no. 1 (2022): 4652, 10.1038/s41467-022-32326-9. PMC9399252 35999195

[34] A. K. Michael , L. Stoos , P. Crosby , et al., “Cooperation Between bHLH Transcription Factors and Histones for DNA Access,” Nature 619, no. 7969 (2023): 385–393, 10.1038/s41586-023-06282-3. PMC10338342 37407816

[35] T. Sato and C. M. Greco , “Expanding the Link Between Circadian Rhythms and Redox Metabolism of Epigenetic Control,” Free Radical Biology and Medicine 170 (2021): 50–58, 10.1016/j.freeradbiomed.2021.01.009. 33450380

[36] C. A. Wolff , M. A. Gutierrez‐Monreal , L. Meng , et al., “Defining the Age‐Dependent and Tissue‐Specific Circadian Transcriptome in Male Mice,” Cell Reports 42, no. 1 (2023): 111982, 10.1016/j.celrep.2022.111982. PMC9929559 36640301

[37] C. H. Gabriel , M. Del Olmo , A. Zehtabian , et al., “Live‐Cell Imaging of Circadian Clock Protein Dynamics in CRISPR‐Generated Knock‐In Cells,” Nature Communications 12, no. 1 (2021): 3796, 10.1038/s41467-021-24086-9. PMC8213786 34145278

[38] Y. Zhao , Y. Li , T. Lu , et al., “Research Progress on the Role and Mechanism of Circadian Clock Gene PER1 in the Occurrence and Development of Multiple Diseases,” Frontiers in Immunology 16 (2025): 1715457, 10.3389/fimmu.2025.1715457. PMC12727441 41451215

[39] X. Cao , Y. Yang , C. P. Selby , Z. Liu , and A. Sancar , “Molecular Mechanism of the Repressive Phase of the Mammalian Circadian Clock,” Proceedings of the National Academy of Sciences of the United States of America 118, no. 2 (2021): e2021174118, 10.1073/pnas.2021174118. PMC7812753 33443219

[40] Y. Otobe , E. M. Jeong , S. Ito , et al., “Phosphorylation of DNA‐Binding Domains of CLOCK‐BMAL1 Complex for PER‐Dependent Inhibition in Circadian Clock of Mammalian Cells,” Proceedings of the National Academy of Sciences of the United States of America 121, no. 23 (2024): e2316858121, 10.1073/pnas.2316858121. PMC11161756 38805270

[41] M. Adlanmerini and M. A. Lazar , “The REV‐ERB Nuclear Receptors: Timekeepers for the Core Clock Period and Metabolism,” Endocrinology 164, no. 6 (2023): bqad069, 10.1210/endocr/bqad069. PMC10413432 37149727

[42] Q. Wang , I. K. Sundar , J. H. Lucas , et al., “Circadian Clock Molecule REV‐ERBα Regulates Lung Fibrotic Progression Through Collagen Stabilization,” Nature Communications 14, no. 1 (2023): 1295, 10.1038/s41467-023-36896-0. PMC9996598 36894533

[43] T. Sato and P. Sassone‐Corsi , “Nutrition, Metabolism, and Epigenetics: Pathways of Circadian Reprogramming,” EMBO Reports 23, no. 5 (2022): e52412, 10.15252/embr.202152412. PMC9066069 35412705

[44] C. Zhang , L. Chen , L. Sun , et al., “BMAL1 Collaborates With CLOCK to Directly Promote DNA Double‐Strand Break Repair and Tumor Chemoresistance,” Oncogene 42, no. 13 (2023): 967–979, 10.1038/s41388-023-02603-y. PMC10038804 36725890

[45] S. K. Soni , P. Basu , M. Singaravel , et al., “Sirtuins and the Circadian Clock Interplay in Cardioprotection: Focus on Sirtuin 1,” Cellular and Molecular Life Sciences 78, no. 6 (2021): 2503–2515, 10.1007/s00018-020-03713-6. PMC11073088 33388853

[46] J. A. Ritonja , K. J. Aronson , L. Flaten , et al., “Exploring the Impact of Night Shift Work on Methylation of Circadian Genes,” Epigenetics 17, no. 10 (2022): 1259–1268, 10.1080/15592294.2021.2009997. PMC9542576 34825628

[47] J. D. Thacher , A. Snigireva , U. M. Dauter , et al., “Road Traffic Noise and Breast Cancer: DNA Methylation in Four Core Circadian Genes,” Clinical Epigenetics 16, no. 1 (2024): 168, 10.1186/s13148-024-01774-z. PMC11590349 39587706

[48] Y. Zhu , Y. Zheng , R. Dai , and X. Gu , “Crosstalk Between Circadian Rhythm Dysregulation and Tumorigenesis, Tumor Metabolism and Tumor Immune Response,” Aging and Disease 16, no. 4 (2024): 2073–2099, 10.14336/AD.2024.0533. PMC12221385 39012661

[49] M. Ye , Y. Lin , S. Pan , Z. W. Wang , and X. Zhu , “Applications of Multi‐Omics Approaches for Exploring the Molecular Mechanism of Ovarian Carcinogenesis,” Frontiers in Oncology 11 (2021): 745808, 10.3389/fonc.2021.745808. PMC8497990 34631583

[50] J. Grau , F. Schmidt , and M. H. Schulz , “Widespread Effects of DNA Methylation and Intra‐Motif Dependencies Revealed by Novel Transcription Factor Binding Models,” Nucleic Acids Research 51, no. 18 (2023): e95, 10.1093/nar/gkad693. PMC10570048 37650641

[51] Y. M. Vuu , C. T. Roberts , and M. Rastegar , “MeCP2 Is an Epigenetic Factor That Links DNA Methylation With Brain Metabolism,” International Journal of Molecular Sciences 24, no. 4 (2023): 4218, 10.3390/ijms24044218. PMC9966807 36835623

[52] L. Saad , J. Zwiller , A. Kalsbeek , and P. Anglard , “Epigenetic Regulation of Circadian Clocks and Its Involvement in Drug Addiction,” Genes 12, no. 8 (2021): 1263, 10.3390/genes12081263. PMC8394526 34440437

[53] X. de Martin , R. Sodaei , and G. Santpere , “Mechanisms of Binding Specificity Among bHLH Transcription Factors,” International Journal of Molecular Sciences 22, no. 17 (2021): 9150, 10.3390/ijms22179150. PMC8431614 34502060

[54] K. J. Tonsfeldt , P. L. Mellon , and H. M. Hoffmann , “Circadian Rhythms in the Neuronal Network Timing the Luteinizing Hormone Surge,” Endocrinology 163, no. 2 (2022): bqab268, 10.1210/endocr/bqab268. PMC8782605 34967900

[55] Y. Jiang , S. Li , W. Xu , et al., “Critical Roles of the Circadian Transcription Factor BMAL1 in Reproductive Endocrinology and Fertility,” Front Endocrinol (Lausanne) 13 (2022): 818272, 10.3389/fendo.2022.818272. PMC8924658 35311235

[56] N. Nakao , S. Yasuo , A. Nishimura , et al., “Circadian Clock Gene Regulation of Steroidogenic Acute Regulatory Protein Gene Expression in Preovulatory Ovarian Follicles,” Endocrinology 148, no. 7 (2007): 3031–3038, 10.1210/en.2007-0044. 17431006

[57] T. Kawamura , Y. Dai , M. Ono , et al., “BMAL1 Positively Correlates With Genes Regulating Steroidogenesis in Human Luteinized Granulosa Cells,” Reproduction 167, no. 2 (2024): e230225, 10.1530/REP-23-0225. 38271820

[58] V. M. Alvord , E. J. Kantra , and J. S. Pendergast , “Estrogens and the Circadian System,” Seminars in Cell and Developmental Biology 126 (2022): 56–65, 10.1016/j.semcdb.2021.04.010. PMC8573061 33975754

[59] H. Valera , A. Chen , and K. J. Grive , “The Hypothalamic‐Pituitary‐Ovarian Axis, Ovarian Disorders, and Brain Aging,” Endocrinology 166, no. 10 (2025): bqaf137, 10.1210/endocr/bqaf137. PMC12448947 40884186

[60] S. Shao , H. Zhao , Z. Lu , X. Lei , and Y. Zhang , “Circadian Rhythms Within the Female HPG Axis: From Physiology to Etiology,” Endocrinology 162, no. 8 (2021): bqab117, 10.1210/endocr/bqab117. PMC8256628 34125877

[61] B. B. Jamieson , G. T. Bouwer , R. E. Campbell , and R. Piet , “Estrous Cycle Plasticity in the Central Clock Output to Kisspeptin Neurons: Implications for the Preovulatory Surge,” Endocrinology 162, no. 6 (2021): bqab071, 10.1210/endocr/bqab071. 33824970

[62] S. Constantin , C. Quignon , K. Pizano , D. M. Shostak , and S. Wray , “Vasoactive Intestinal Peptide Excites GnRH Neurons via KCa3.1, a Potential Player in the Slow Afterhyperpolarization Current,” Frontiers in Cellular Neuroscience 18 (2024): 1354095, 10.3389/fncel.2024.1354095. PMC11021707 38633445

[63] M. Ono , H. Ando , T. Daikoku , et al., “The Circadian Clock, Nutritional Signals and Reproduction: A Close Relationship,” International Journal of Molecular Sciences 24, no. 2 (2023): 1545, 10.3390/ijms24021545. PMC9865912 36675058

[64] G. Beroukhim , E. Esencan , and D. B. Seifer , “Impact of Sleep Patterns Upon Female Neuroendocrinology and Reproductive Outcomes: A Comprehensive Review,” Reproductive Biology and Endocrinology 20, no. 1 (2022): 16, 10.1186/s12958-022-00889-3. PMC8764829 35042515

[65] A. Brzezinski , S. Rai , A. Purohit , and S. R. Pandi‐Perumal , “Melatonin, Clock Genes, and Mammalian Reproduction: What Is the Link?,” International Journal of Molecular Sciences 22, no. 24 (2021): 13240, 10.3390/ijms222413240. PMC8704059 34948038

[66] A. L. Mereness , Z. C. Murphy , A. C. Forrestel , et al., “Conditional Deletion of Bmal1 in Ovarian Theca Cells Disrupts Ovulation in Female Mice,” Endocrinology 157, no. 2 (2016): 913–927, 10.1210/en.2015-1645. PMC5393362 26671182

[67] Y. Zhou , X. He , Z. Chen , et al., “Role of Circadian Clock in Female Embryo Implantation,” Frontiers in Cell and Developmental Biology 13 (2025): 1607491, 10.3389/fcell.2025.1607491. PMC12174376 40535570

[68] D. Varga , P. Szatmári , and E. Ducza , “Inflammatory and Redox Mediators in Rat and Human Ovulation,” International Journal of Molecular Sciences 26, no. 24 (2025): 11979, 10.3390/ijms262411979. PMC12732926 41465405

[69] W. J. Murdoch , R. S. Townsend , and A. C. McDonnel , “Ovulation‐Induced DNA Damage in Ovarian Surface Epithelial Cells of Ewes: Prospective Regulatory Mechanisms of Repair/Survival and Apoptosis,” Biology of Reproduction 65, no. 5 (2001): 1417–1424, 10.1095/biolreprod65.5.1417. 11673258

[70] A. Arafa , M. Alhussein , A. Alayyan , et al., “Is Night Shift Work Associated With Ovarian Cancer? A Systematic Review and Meta‐Analysis,” Medical Science 13, no. 4 (2025): 228, 10.3390/medsci13040228. PMC12551028 41133510

[71] Z. Wang , G. Su , Z. Dai , et al., “Circadian Clock Genes Promote Glioma Progression by Affecting Tumour Immune Infiltration and Tumour Cell Proliferation,” Cell Proliferation 54, no. 3 (2021): e12988, 10.1111/cpr.12988. PMC7941241 33442944

[72] M. Chen , L. Zhang , X. Liu , Z. Ma , and L. Lv , “PER1 Is a Prognostic Biomarker and Correlated With Immune Infiltrates in Ovarian Cancer,” Frontiers in Genetics 12 (2021): 697471, 10.3389/fgene.2021.697471. PMC8248530 34220965

[73] F. Battaglin , P. Chan , Y. Pan , et al., “Clocking Cancer: The Circadian Clock as a Target in Cancer Therapy,” Oncogene 40, no. 18 (2021): 3187–3200, 10.1038/s41388-021-01778-6. PMC8549632 33846572

[74] H. Liu , Y. Liu , R. Hai , W. Liao , and X. Luo , “The Role of Circadian Clocks in Cancer: Mechanisms and Clinical Implications,” Genes and Diseases 10, no. 4 (2022): 1279–1290, 10.1016/j.gendis.2022.05.014. PMC10310980 37397565

[75] L. Losi , S. Fonda , S. Saponaro , et al., “Distinct DNA Methylation Profiles in Ovarian Tumors: Opportunities for Novel Biomarkers,” International Journal of Molecular Sciences 19, no. 6 (2018): 1559, 10.3390/ijms19061559. PMC6032431 29882921

[76] B. J. Altman , A. L. Hsieh , A. Sengupta , et al., “MYC Disrupts the Circadian Clock and Metabolism in Cancer Cells,” Cell Metabolism 22, no. 6 (2015): 1009–1019, 10.1016/j.cmet.2015.09.003. PMC4818967 26387865

[77] F. Sartor , B. Ferrero‐Bordera , J. Haspel , M. Sperandio , P. M. Holloway , and M. Merrow , “Circadian Clock and Hypoxia,” Circulation Research 134, no. 6 (2024): 618–634, 10.1161/CIRCRESAHA.124.323518. 38484033

[78] X. Zheng , X. Wang , X. Cheng , et al., “Single‐Cell Analyses Implicate Ascites in Remodeling the Ecosystems of Primary and Metastatic Tumors in Ovarian Cancer,” Nature Cancer 4, no. 8 (2023): 1138–1156, 10.1038/s43018-023-00599-8. PMC10447252 37488416

[79] N. Rembiałkowska , K. Rekiel , P. Urbanowicz , et al., “Epigenetic Dysregulation in Cancer: Implications for Gene Expression and DNA Repair‐Associated Pathways,” International Journal of Molecular Sciences 26, no. 13 (2025): 6531, 10.3390/ijms26136531. PMC12249909 40650308

[80] H. Romero , A. Amiri , M. K. Pabba , et al., “MeCP2‐Driven Chromatin Organization Controls Nuclear Stiffness,” Communications Biology 9, no. 1 (2025): 60, 10.1038/s42003-025-09328-6. PMC12804834 41360929

[81] N. Nelson , O. Zimmer , and A. Relógio , “Personalized Chronotherapy in Glioblastoma: Integrating Circadian Profiling and PK‐PD Modelling to Optimize Temozolomide Timing,” NPJ Precision Oncology 10, no. 1 (2025): 17, 10.1038/s41698-025-01205-z. PMC12796165 41387951

[82] Z. Wang , F. Li , M. Wei , S. Zhang , and T. Wang , “Circadian Clock Protein PERIOD2 Suppresses the PI3K/Akt Pathway and Promotes Cisplatin Sensitivity in Ovarian Cancer,” Cancer Management and Research 12 (2020): 11897–11908, 10.2147/CMAR.S278903. PMC7683831 33244267

[83] R. J. Lund , K. Huhtinen , J. Salmi , et al., “DNA Methylation and Transcriptome Changes Associated With Cisplatin Resistance in Ovarian Cancer,” Scientific Reports 7, no. 1 (2017): 1469, 10.1038/s41598-017-01624-4. PMC5431431 28473707

[84] Y. Zheng , G. Huang , T. C. Silva , et al., “A Pan‐Cancer Analysis of CpG Island Gene Regulation Reveals Extensive Plasticity Within Polycomb Target Genes,” Nature Communications 12, no. 1 (2021): 2485, 10.1038/s41467-021-22720-0. PMC8087678 33931649

[85] E. Biskup , J. Lopacinska‐Jørgensen , L. K. Vestergaard , and E. Høgdall , “Validating Reference‐Based Algorithms to Determine Cell‐Type Heterogeneity in Ovarian Cancer DNA Methylation Studies,” Scientific Reports 14, no. 1 (2024): 11048, 10.1038/s41598-024-61857-y. PMC11094148 38745057

[86] D. Sharma and C. L. Partch , “PAS Dimerization at the Nexus of the Mammalian Circadian Clock,” Journal of Molecular Biology 436, no. 3 (2024): 168341, 10.1016/j.jmb.2023.168341. PMC11729053 37924861

[87] J. Jin , T. Lian , C. Gu , K. Yu , Y. Q. Gao , and X. D. Su , “The Effects of Cytosine Methylation on General Transcription Factors,” Scientific Reports 6 (2016): 29119, 10.1038/srep29119. PMC4935894 27385050

[88] C. Alkhoury , N. F. Henneman , V. Petrenko , et al., “Class 3 PI3K Coactivates the Circadian Clock to Promote Rhythmic de Novo Purine Synthesis,” Nature Cell Biology 25, no. 7 (2023): 975–988, 10.1038/s41556-023-01171-3. PMC10344785 37414850

[89] R. M. Mello , S. Masri , and K. A. Lamia , “Rhythms of Risk: The Intersection of Clocks, Cancer, and Chronotherapy,” Journal of Clinical Investigation 136, no. 3 (2026): e198780, 10.1172/JCI198780. PMC12867155 41623166

[90] F. C. Martins , D. L. Couturier , I. de Santiago , et al., “Clonal Somatic Copy Number Altered Driver Events Inform Drug Sensitivity in High‐Grade Serous Ovarian Cancer,” Nature Communications 13, no. 1 (2022): 6360, 10.1038/s41467-022-33870-0. PMC9606297 36289203

[91] A. Panigrahi and B. W. O'Malley , “Mechanisms of Enhancer Action: The Known and the Unknown,” Genome Biology 22, no. 1 (2021): 108, 10.1186/s13059-021-02322-1. PMC8051032 33858480

[92] A. Bafna , G. Banks , M. H. Hastings , and P. M. Nolan , “Dynamic Modulation of Genomic Enhancer Elements in the Suprachiasmatic Nucleus, the Site of the Mammalian Circadian Clock,” Genome Research 33, no. 5 (2023): 673–688, 10.1101/gr.277581.122. PMC10317116 37156620

[93] Y. Bi , K. Zou , and K. Wang , “Super‐Enhancer DNA Methylation in Cancer: The Mechanism of Action and Therapeutic Directions,” Frontiers in Oncology 15 (2025): 1610579, 10.3389/fonc.2025.1610579. PMC12520912 41103952

[94] S. Kaluscha , S. Domcke , C. Wirbelauer , et al., “Evidence That Direct Inhibition of Transcription Factor Binding Is the Prevailing Mode of Gene and Repeat Repression by DNA Methylation,” Nature Genetics 54, no. 12 (2022): 1895–1906, 10.1038/s41588-022-01241-6. PMC9729108 36471082

[95] A. Monteagudo‐Sánchez , J. Richard Albert , M. Scarpa , D. Noordermeer , and M. V. C. Greenberg , “The Impact of the Embryonic DNA Methylation Program on CTCF‐Mediated Genome Regulation,” Nucleic Acids Research 52, no. 18 (2024): 10934–10950, 10.1093/nar/gkae724. PMC11472158 39180406

[96] L. N. Guerin , T. J. Scott , J. A. Yap , et al., “Temporally Discordant Chromatin Accessibility and DNA Demethylation Define Short‐ and Long‐Term Enhancer Regulation During Cell Fate Specification,” Cell Reports 44, no. 5 (2025): 115680, 10.1016/j.celrep.2025.115680. PMC12153380 40349339

[97] M. Ahmed , F. Soares , J. H. Xia , et al., “CRISPRi Screens Reveal a DNA Methylation‐Mediated 3D Genome Dependent Causal Mechanism in Prostate Cancer,” Nature Communications 12, no. 1 (2021): 1781, 10.1038/s41467-021-21867-0. PMC7979745 33741908

[98] S. A. Roseman , A. P. Siegenfeld , C. Lee , N. Z. Lue , A. L. Waterbury , and B. B. Liau , “DNA Methylation Insulates Genic Regions From CTCF Loops Near Nuclear Speckles,” eLife 13 (2025): RP102930, 10.7554/eLife.102930. PMC12408068 40900123

[99] S. Shang , J. Yang , A. A. Jazaeri , et al., “Chemotherapy‐Induced Distal Enhancers Drive Transcriptional Programs to Maintain the Chemoresistant State in Ovarian Cancer,” Cancer Research 79, no. 18 (2019): 4599–4611, 10.1158/0008-5472.CAN-19-0215. PMC6744966 31358529

[100] J. Gallon , E. Loomis , E. Curry , et al., “Chromatin Accessibility Changes at Intergenic Regions Are Associated With Ovarian Cancer Drug Resistance,” Clinical Epigenetics 13, no. 1 (2021): 122, 10.1186/s13148-021-01105-6. PMC8180030 34090482

[101] J. R. Beytebiere , A. J. Trott , B. J. Greenwell , et al., “Tissue‐Specific BMAL1 Cistromes Reveal That Rhythmic Transcription Is Associated With Rhythmic Enhancer‐Enhancer Interactions,” Genes and Development 33, no. 5–6 (2019): 294–309, 10.1101/gad.322198.118. PMC6411008 30804225

[102] K. H. Cox and J. S. Takahashi , “Circadian Clock Genes and the Transcriptional Architecture of the Clock Mechanism,” Journal of Molecular Endocrinology 63, no. 4 (2019): R93–R102, 10.1530/JME-19-0153. PMC6872945 31557726

[103] P. Bhatti , K. L. Cushing‐Haugen , K. G. Wicklund , J. A. Doherty , and M. A. Rossing , “Nightshift Work and Risk of Ovarian Cancer,” Occupational and Environmental Medicine 70, no. 4 (2013): 231–237, 10.1136/oemed-2012-101146. PMC3777774 23343856

[104] K. B. Megha , A. Arathi , S. Shikha , R. Alka , P. Ramya , and P. V. Mohanan , “Significance of Melatonin in the Regulation of Circadian Rhythms and Disease Management,” Molecular Neurobiology 61, no. 8 (2024): 5541–5571, 10.1007/s12035-024-03915-0. 38206471

[105] M. A. Bonmati‐Carrion and A. Tomas‐Loba , “Melatonin and Cancer: A Polyhedral Network Where the Source Matters,” Antioxidants (Basel) 10, no. 2 (2021): 210, 10.3390/antiox10020210. PMC7912767 33535472

[106] M. Zhao , J. Wan , K. Zeng , et al., “The Reduction in Circulating Melatonin Level May Contribute to the Pathogenesis of Ovarian Cancer: A Retrospective Study,” Journal of Cancer 7, no. 7 (2016): 831–836, 10.7150/jca.14573. PMC4860800 27162542

[107] M. S. Cucielo , P. P. Freire , M. T. Emílio‐Silva , et al., “Melatonin Enhances Cell Death and Suppresses the Metastatic Capacity of Ovarian Cancer Cells by Attenuating the Signaling of Multiple Kinases,” Pathology, Research and Practice 248 (2023): 154637, 10.1016/j.prp.2023.154637. 37356221

[108] M. S. Cucielo , R. C. Cesário , H. S. Silveira , et al., “Melatonin Reverses the Warburg‐Type Metabolism and Reduces Mitochondrial Membrane Potential of Ovarian Cancer Cells Independent of MT1 Receptor Activation,” Molecules 27, no. 14 (2022): 4350, 10.3390/molecules27144350. PMC9321770 35889222

[109] H. S. Silveira , R. C. Cesário , R. A. Vígaro , et al., “Melatonin Changes Energy Metabolism and Reduces Oncogenic Signaling in Ovarian Cancer Cells,” Molecular and Cellular Endocrinology 592 (2024): 112296, 10.1016/j.mce.2024.112296. 38844096

[110] S. Bu , Q. Wang , J. Sun , X. Li , T. Gu , and D. Lai , “Melatonin Suppresses Chronic Restraint Stress‐Mediated Metastasis of Epithelial Ovarian Cancer via NE/AKT/β‐Catenin/SLUG Axis,” Cell Death and Disease 11, no. 8 (2020): 644, 10.1038/s41419-020-02906-y. PMC7435194 32811805

[111] K. Linowiecka , A. T. Slominski , R. J. Reiter , et al., “Melatonin: A Potential Regulator of DNA Methylation,” Antioxidants 12, no. 6 (2023): 1155, 10.3390/antiox12061155. PMC10295183 37371885

[112] A. Davoodvandi , B. Nikfar , R. J. Reiter , and Z. Asemi , “Melatonin and Cancer Suppression: Insights Into Its Effects on DNA Methylation,” Cellular and Molecular Biology Letters 27, no. 1 (2022): 73, 10.1186/s11658-022-00375-z. PMC9446540 36064311

[113] J. Bautista , S. Ojeda‐Mosquera , D. Ordóñez‐Lozada , and A. López‐Cortés , “Peripheral Clocks and Systemic Zeitgeber Interactions: From Molecular Mechanisms to Circadian Precision Medicine,” Front Endocrinol (Lausanne) 16 (2025): 1606242, 10.3389/fendo.2025.1606242. PMC12158691 40510487

[114] I. Chihara , H. Negoro , J. Kono , et al., “Glucocorticoids Coordinate the Bladder Peripheral Clock and Diurnal Micturition Pattern in Mice,” Communications Biology 6, no. 1 (2023): 81, 10.1038/s42003-023-04464-3. PMC9867708 36681730

[115] A. Balsalobre , S. A. Brown , L. Marcacci , et al., “Resetting of Circadian Time in Peripheral Tissues by Glucocorticoid Signaling,” Science 289, no. 5488 (2000): 2344–2347, 10.1126/science.289.5488.2344. 11009419

[116] M. Cuesta , N. Cermakian , and D. B. Boivin , “Glucocorticoids Entrain Molecular Clock Components in Human Peripheral Cells,” FASEB Journal 29, no. 4 (2015): 1360–1370, 10.1096/fj.14-265686. 25500935

[117] L. Zillich , N. S. Czernin , O. C. Salvador , et al., “Acute Stress Induces Changes in Epigenome‐Wide DNA Methylation,” Neuropsychopharmacology 51, no. 4 (2026): 732–740, 10.1038/s41386-025-02289-8. PMC12932828 41387570

[118] R. Buonaiuto , G. Neola , S. C. Cecere , et al., “Glucocorticoid Receptor and Ovarian Cancer: From Biology to Therapeutic Intervention,” Biomolecules 13, no. 4 (2023): 653, 10.3390/biom13040653. PMC10135974 37189400

[119] J. Bakusic , M. Ghosh , A. Polli , et al., “Role of NR3C1 and SLC6A4 Methylation in the HPA Axis Regulation in Burnout,” Journal of Affective Disorders 295 (2021): 505–512, 10.1016/j.jad.2021.08.081. 34509065

[120] J. de Assis Pinheiro , F. V. Freitas , A. R. Borçoi , et al., “Alcohol Consumption, Depression, Overweight and Cortisol Levels as Determining Factors for NR3C1 Gene Methylation,” Scientific Reports 11, no. 1 (2021): 6768, 10.1038/s41598-021-86189-z. PMC7990967 33762648

[121] K. Yagita , “Emergence of the Circadian Clock Oscillation During the Developmental Process in Mammals,” Current Opinion in Genetics and Development 84 (2024): 102152, 10.1016/j.gde.2024.102152. 38266394

[122] Y. Otobe and H. Yoshitane , “Phosphorylation of CLOCK and BMAL1‐a Key Regulatory Mechanism in the Mammalian Circadian Clockwork,” FEBS Letters 600, no. 6 (2025): 732–737, 10.1002/1873-3468.70194. 41108717

[123] H. Mok , E. Ostendorf , A. Ganninger , A. J. Adler , G. Hazan , and J. A. Haspel , “Circadian Immunity From Bench to Bedside: A Practical Guide,” Journal of Clinical Investigation 134, no. 3 (2024): e175706, 10.1172/JCI175706. PMC10836804 38299593

[124] Y. Yuan , Q. Chen , M. Brovkina , E. J. Clowney , and S. Yadlapalli , “Clock‐Dependent Chromatin Accessibility Rhythms Regulate Circadian Transcription,” PLoS Genetics 20, no. 5 (2024): e1011278, 10.1371/journal.pgen.1011278. PMC11161047 38805552

[125] D. Halawani , Y. Wang , A. Ramakrishnan , et al., “Circadian Clock Regulator Bmal1 Gates Axon Regeneration via Tet3 Epigenetics in Mouse Sensory Neurons,” Nature Communications 14, no. 1 (2023): 5165, 10.1038/s41467-023-40816-7. PMC10449865 37620297

[126] A. Szredzka , A. Chwastowicz , J. Pergoł , and P. Matryba , “Circadian Clocks and Adaptive Immune Function: From Mechanisms to Therapeutic Applications,” Frontiers in Immunology 16 (2025): 1697854, 10.3389/fimmu.2025.1697854. PMC12708332 41415271

[127] M. Turpin and G. Salbert , “5‐Methylcytosine Turnover: Mechanisms and Therapeutic Implications in Cancer,” Frontiers in Molecular Biosciences 9 (2022): 976862, 10.3389/fmolb.2022.976862. PMC9428128 36060265

[128] L. Zhu and Q. Zhou , “Aberrant Epigenetic Regulation of FZD3 by TET2 Is Involved in Ovarian Cancer Cell Resistance to Cisplatin,” Journal of Chemotherapy 36, no. 2 (2024): 143–155, 10.1080/1120009X.2023.2219920. 37300277

[129] S. Y. Yang , J. S. Jhang , W. L. Huang , et al., “Wogonin Inhibits Ovarian Cancer by Activating the AMPK‐TET2‐5hmC Axis,” Molecular Carcinogenesis 64, no. 3 (2025): 440–449, 10.1002/mc.23856. 39620386

[130] M. Weigert , X. L. Cui , D. West‐Szymanski , et al., “5‐Hydroxymethylcytosine Signals in Serum Are a Predictor of Chemoresistance in High‐Grade Serous Ovarian Cancer,” Gynecologic Oncology 182 (2024): 82–90, 10.1016/j.ygyno.2024.01.001. PMC11246748 38262243

[131] F. Wang , L. Wang , Z. Xing , et al., “Multi‐Analyte Approach Combining cfDNA Sequencing and Protein Testing for Early Ovarian Cancer Detection,” IScience 28, no. 6 (2025): 112617, 10.1016/j.isci.2025.112617. PMC12152359 40502685

[132] Y. Wang , H. Xie , X. Chang , et al., “Single‐Cell Dissection of the Multiomic Landscape of High‐Grade Serous Ovarian Cancer,” Cancer Research 82, no. 21 (2022): 3903–3916, 10.1158/0008-5472.CAN-21-3819. PMC9627134 35969151

[133] R. Meddeb , E. Pisareva , and A. R. Thierry , “Guidelines for the Preanalytical Conditions for Analyzing Circulating Cell‐Free DNA,” Clinical Chemistry 65, no. 5 (2019): 623–633, 10.1373/clinchem.2018.298323. 30792266

[134] J. Wang , J. Li , R. Chen , et al., “DNA Methylation‐Based Profiling Reveals Distinct Clusters With Survival Heterogeneity in High‐Grade Serous Ovarian Cancer,” Clinical Epigenetics 13, no. 1 (2021): 190, 10.1186/s13148-021-01178-3. PMC8515755 34645493

[135] G. S. Collins , J. B. Reitsma , D. G. Altman , and K. G. Moons , “Transparent Reporting of a Multivariable Prediction Model for Individual Prognosis or Diagnosis (TRIPOD): The TRIPOD Statement,” Annals of Internal Medicine 162, no. 1 (2015): 55–63, 10.7326/M14-0697. 25560714

[136] M. J. Rutten , J. H. Boldingh , E. Schuit , et al., “Development and Internal Validation of a Prognostic Model for Survival After Debulking Surgery for Epithelial Ovarian Cancer,” Gynecologic Oncology 135, no. 1 (2014): 13–18, 10.1016/j.ygyno.2014.07.099. 25093289

[137] J. Shilts , G. Chen , and J. J. Hughey , “Evidence for Widespread Dysregulation of Circadian Clock Progression in Human Cancer,” PeerJ 6 (2018): e4327, 10.7717/peerj.4327. PMC5797448 29404219

[138] S. Nag , I. Ray Coquard , S. Gupta , et al., “Pan‐Asia Adapted ESMO Clinical Practice Guideline for the Management of Patients With Newly Diagnosed and Relapsed Epithelial Ovarian Cancer,” ESMO Open 10, no. 6 (2025): 105125, 10.1016/j.esmoop.2025.105125. PMC12180976 40494042

[139] P. L. Riso , C. E. Villa , G. Gasparoni , et al., “A Cell‐Of‐Origin Epigenetic Tracer Reveals Clinically Distinct Subtypes of High‐Grade Serous Ovarian Cancer,” Genome Medicine 12, no. 1 (2020): 94, 10.1186/s13073-020-00786-7. PMC7597028 33121525

[140] L. Zhao , Y. Tang , J. Yang , et al., “Integrative Analysis of Circadian Clock With Prognostic and Immunological Biomarker Identification in Ovarian Cancer,” Frontiers in Molecular Biosciences 10 (2023): 1208132, 10.3389/fmolb.2023.1208132. PMC10318361 37409345

[141] X. Xing , F. Gu , L. Hua , et al., “TIMELESS Promotes Tumor Progression by Enhancing Macrophages Recruitment in Ovarian Cancer,” Frontiers in Oncology 11 (2021): 732058, 10.3389/fonc.2021.732058. PMC8417241 34490127

[142] D. B. Asante , D. Tierno , G. Grassi , and B. Scaggiante , “Circulating Tumour DNA for Ovarian Cancer Diagnosis and Treatment Monitoring: What Perspectives for Clinical Use?,” International Journal of Molecular Sciences 26, no. 5 (2025): 1889, 10.3390/ijms26051889. PMC11900478 40076521

[143] Y. Zhang , Y. Guan , X. Xiao , et al., “Circulating Tumor DNA Detection Improves Relapse Prediction in Epithelial Ovarian Cancer,” BMC Cancer 24, no. 1 (2024): 1565, 10.1186/s12885-024-13222-5. PMC11663307 39710659

[144] Y. M. Kim , S. W. Lee , Y. J. Lee , H. Y. Lee , J. E. Lee , and E. K. Choi , “Prospective Study of the Efficacy and Utility of TP53 Mutations in Circulating Tumor DNA as a Non‐Invasive Biomarker of Treatment Response Monitoring in Patients With High‐Grade Serous Ovarian Carcinoma,” Journal of Gynecologic Oncology 30, no. 3 (2019): e32, 10.3802/jgo.2019.30.e32. PMC6424844 30887755

[145] Cancer Genome Atlas Research Network , “Integrated Genomic Analyses of Ovarian Carcinoma,” Nature 474, no. 7353 (2011): 609–615, 10.1038/nature10166. PMC3163504 21720365

[146] H. Xu , Z. Wang , G. Mo , and H. Chen , “Association Between Circadian Gene CLOCK and Cisplatin Resistance in Ovarian Cancer Cells: A Preliminary Study,” Oncology Letters 15, no. 6 (2018): 8945–8950, 10.3892/ol.2018.8488. PMC5958788 29844814

[147] L. Wang , X. Wang , X. Zhu , et al., “Drug Resistance in Ovarian Cancer: From Mechanism to Clinical Trial,” Molecular Cancer 23, no. 1 (2024): 66, 10.1186/s12943-024-01967-3. PMC10976737 38539161

[148] C. Zhao , Y. Liu , and Z. Cui , “Epigenetic Regulation in Gynecological Cancers: A Paradigm Shift in Immunotherapy Strategies,” Journal of Experimental & Clinical Cancer Research 44, no. 1 (2025): 275, 10.1186/s13046-025-03509-1. PMC12487091 41029427

[149] K. B. Chiappinelli and S. B. Baylin , “Inhibiting DNA Methylation Improves Antitumor Immunity in Ovarian Cancer,” Journal of Clinical Investigation 132, no. 14 (2022): e160186, 10.1172/JCI160186. PMC9282922 35838045

[150] A. M. Oza , U. A. Matulonis , A. Alvarez Secord , et al., “A Randomized Phase II Trial of Epigenetic Priming With Guadecitabine and Carboplatin in Platinum‐Resistant, Recurrent Ovarian Cancer,” Clinical Cancer Research 26, no. 5 (2020): 1009–1016, 10.1158/1078-0432.CCR-19-1638. PMC7056559 31831561

[151] D. Matei , S. Ghamande , L. Roman , et al., “A Phase I Clinical Trial of Guadecitabine and Carboplatin in Platinum‐Resistant, Recurrent Ovarian Cancer: Clinical, Pharmacokinetic, and Pharmacodynamic Analyses,” Clinical Cancer Research 24, no. 10 (2018): 2285–2293, 10.1158/1078-0432.CCR-17-3055. PMC5955794 29500276

[152] B. V. Landon , J. L. Boland , A. E. Wahner Hendrickson , et al., “Pembrolizumab and Epigenetic Modification With Azacitidine Reshapes the Tumor Microenvironment of Platinum‐Resistant Epithelial Ovarian Cancer: A Phase 2 Non‐Randomized Clinical Trial,” Communications Medicine 6 (2026): 142, 10.1038/s43856-026-01404-0. PMC12988192 41652014

[153] H. Meteran , A. Ø. Knudsen , T. L. Jørgensen , D. Nielsen , and J. Herrstedt , “Carboplatin Plus Paclitaxel in Combination With the Histone Deacetylate Inhibitor, Vorinostat, in Patients With Recurrent Platinum‐Sensitive Ovarian Cancer,” Journal of Clinical Medicine 13, no. 3 (2024): 897, 10.3390/jcm13030897. PMC10856581 38337591

[154] A. Amiama‐Roig , E. M. Verdugo‐Sivianes , A. Carnero , and J. R. Blanco , “Chronotherapy: Circadian Rhythms and Their Influence in Cancer Therapy,” Cancers 14, no. 20 (2022): 5071, 10.3390/cancers14205071. PMC9599830 36291855

[155] M. I. Printezi , A. B. Kilgallen , M. J. G. Bond , et al., “Toxicity and Efficacy of Chronomodulated Chemotherapy: A Systematic Review,” Lancet Oncology 23, no. 3 (2022): e129–e143, 10.1016/S1470-2045(21)00639-2. 35240088

[156] J. Zhou , J. Wang , X. Zhang , and Q. Tang , “New Insights Into Cancer Chronotherapies,” Frontiers in Pharmacology 12 (2021): 741295. Published 2021 Dec 13, 10.3389/fphar.2021.741295. PMC8710512 34966277

[157] W. J. Hrushesky , “Circadian Timing of Cancer Chemotherapy,” Science 228, no. 4695 (1985): 73–75, 10.1126/science.3883493. 3883493

[158] F. Lévi , M. Benavides , C. Chevelle , et al., “Chemotherapy of Advanced Ovarian Cancer With 4'‐O‐Tetrahydropyranyl Doxorubicin and Cisplatin: A Randomized Phase II Trial With an Evaluation of Circadian Timing and Dose‐Intensity,” Journal of Clinical Oncology 8, no. 4 (1990): 705–714, 10.1200/JCO.1990.8.4.705. 2179481

[159] A. Romero‐Franco , C. Checa‐Rodríguez , S. Jimeno , et al., “Circadian Regulation of Homologous Recombination by cryptochrome1‐Mediated Dampening of DNA End Resection,” Nature Communications 16, no. 1 (2025): 10802, 10.1038/s41467-025-65854-1. PMC12669580 41326346

[160] L. Nougarede , F. Hazane‐Puch , F. de Fraipont , E. Jacquet , and M. Bidart , “BRCA1 Promoter Methylation Predicts PARPi Response in Ovarian Cancer: Insights From the KOMET Study,” Clinical Epigenetics 17, no. 1 (2025): 140, 10.1186/s13148-025-01917-w. PMC12329904 40775791

[161] O. Kondrashova , M. Topp , K. Nesic , et al., “Methylation of All BRCA1 Copies Predicts Response to the PARP Inhibitor Rucaparib in Ovarian Carcinoma,” Nature Communications 9, no. 1 (2018): 3970, 10.1038/s41467-018-05564-z. PMC6162272 30266954

[162] F. Menghi , K. Banda , P. Kumar , et al., “Genomic and Epigenomic BRCA Alterations Predict Adaptive Resistance and Response to Platinum‐Based Therapy in Patients With Triple‐Negative Breast and Ovarian Carcinomas,” Science Translational Medicine 14, no. 652 (2022): eabn1926, 10.1126/scitranslmed.abn1926. PMC9585706 35857626

[163] K. Nesic , O. Kondrashova , R. M. Hurley , et al., “Acquired RAD51C Promoter Methylation Loss Causes PARP Inhibitor Resistance in High‐Grade Serous Ovarian Carcinoma,” Cancer Research 81, no. 18 (2021): 4709–4722, 10.1158/0008-5472.CAN-21-0774. PMC12593227 34321239

[164] R. M. Hurley , C. D. McGehee , K. Nesic , et al., “Characterization of a RAD51C‐Silenced High‐Grade Serous Ovarian Cancer Model During Development of PARP Inhibitor Resistance,” NAR Cancer 3, no. 3 (2021): zcab028, 10.1093/narcan/zcab028. PMC8271218 34316715

[165] M. Bracci , V. Ciarapica , M. E. Zabaleta , et al., “BRCA1 and BRCA2 Gene Expression: Diurnal Variability and Influence of Shift Work,” Cancers 11, no. 8 (2019): 1146, 10.3390/cancers11081146. PMC6721503 31405066

[166] M. Elazezy , K. Prieske , L. Kluwe , et al., “BRCA1 Promoter Hypermethylation on Circulating Tumor DNA Correlates With Improved Survival of Patients With Ovarian Cancer,” Molecular Oncology 15, no. 12 (2021): 3615–3625, 10.1002/1878-0261.13108. PMC8637552 34601813

[167] N. Gull , M. R. Jones , P. C. Peng , et al., “DNA Methylation and Transcriptomic Features Are Preserved Throughout Disease Recurrence and Chemoresistance in High Grade Serous Ovarian Cancers,” Journal of Experimental and Clinical Cancer Research 41, no. 1 (2022): 232, 10.1186/s13046-022-02440-z. PMC9327231 35883104

[168] D. Marri , D. Filipovic , O. Kana , S. Tischkau , and S. Bhattacharya , “Prediction of Mammalian Tissue‐Specific CLOCK‐BMAL1 Binding to E‐Box DNA Motifs,” Scientific Reports 13, no. 1 (2023): 7742, 10.1038/s41598-023-34115-w. PMC10182026 37173345

[169] R. W. Zhou and R. E. Parsons , “Etiology of Super‐Enhancer Reprogramming and Activation in Cancer,” Epigenetics and Chromatin 16, no. 1 (2023): 29, 10.1186/s13072-023-00502-w. PMC10324276 37415185

[170] D. Bisht , A. Arora , and M. Sachan , “Role of DNA De‐Methylation Intermediate ‘5‐Hydroxymethylcytosine’ in Ovarian Cancer Management: A Comprehensive Review,” Biomedicine and Pharmacotherapy 155 (2022): 113674, 10.1016/j.biopha.2022.113674. 36099791

[171] X. Zhang , Y. Zhang , C. Wang , and X. Wang , “TET (Ten‐Eleven Translocation) Family Proteins: Structure, Biological Functions and Applications,” Signal Transduction and Targeted Therapy 8, no. 1 (2023): 297, 10.1038/s41392-023-01537-x. PMC10415333 37563110

[172] O. Morante‐Palacios , L. Ciudad , R. Micheroli , et al., “Coordinated Glucocorticoid Receptor and MAFB Action Induces Tolerogenesis and Epigenome Remodeling in Dendritic Cells,” Nucleic Acids Research 50, no. 1 (2022): 108–126, 10.1093/nar/gkab1182. PMC8754638 34893889

